# The Molecular Chaperone HSPA2 Plays a Key Role in Regulating the Expression of Sperm Surface Receptors That Mediate Sperm-Egg Recognition

**DOI:** 10.1371/journal.pone.0050851

**Published:** 2012-11-29

**Authors:** Kate A. Redgrove, Brett Nixon, Mark A. Baker, Louise Hetherington, Gordon Baker, De-Yi Liu, R. John Aitken

**Affiliations:** 1 Priority Research Centre in Reproductive Science, School of Environmental and Life Sciences, Discipline of Biological Sciences, The University of Newcastle, Callaghan, New South Wales, Australia; 2 Melbourne In Vitro Fertilisation, East Melbourne, Victoria, Australia, and Department of Obstetrics and Gynaecology, The University of Melbourne, Royal Women’s Hospital, Carlton, Victoria, Australia; Clermont-Ferrand Univ., France

## Abstract

A common defect encountered in the spermatozoa of male infertility patients is an idiopathic failure of sperm–egg recognition. In order to resolve the molecular basis of this condition we have compared the proteomic profiles of spermatozoa exhibiting an impaired capacity for sperm-egg recognition with normal cells using label free mass spectrometry (MS)-based quantification. This analysis indicated that impaired sperm–zona binding was associated with reduced expression of the molecular chaperone, heat shock 70 kDa protein 2 (HSPA2), from the sperm proteome. Western blot analysis confirmed this observation in independent patients and demonstrated that the defect did not extend to other members of the HSP70 family. HSPA2 was present in the acrosomal domain of human spermatozoa as a major component of 5 large molecular mass complexes, the most dominant of which was found to contain HSPA2 in close association with just two other proteins, sperm adhesion molecule 1 (SPAM1) and arylsulfatase A (ARSA), both of which that have previously been implicated in sperm-egg interaction. The interaction between SPAM1, ARSA and HSPA2 in a multimeric complex mediating sperm-egg interaction, coupled with the complete failure of this process when HSPA2 is depleted in infertile patients, provides new insights into the mechanisms by which sperm function is impaired in cases of male infertility.

## Introduction

Male infertility is a distressingly common condition affecting at least 1 in 20 men of reproductive age [1]. Apart from the 15% of male patients exhibiting azoospermia [2], [3] a vast majority of infertile men do not suffer from a lack of spermatozoa as much as a loss of sperm function. In this context, one of the most frequently encountered functional defects in these cells is an inability to engage in the complex cascade of cellular processes that enable the spermatozoon to recognize the egg [4]. Despite its biological importance, sperm–egg recognition is still poorly characterized at the molecular level. At insemination more than 100 million sperm cells are released into the female reproductive tract with the task of finding just one other cell in the body, the oocyte. The spermatozoa will make contact with thousands of other cells on their journey from the site of insemination in the lower female reproductive tract to the ampullae of the Fallopian tubes, where fertilization takes place. However, as soon as the sperm plasma membrane makes contact with the zona pellucida (ZP), a unique cell-cell recognition event occurs that triggers a cascade of intercellular interactions leading to fertilization [5]. This event is exquisitely cell specific and very tightly regulated. Freshly ejaculated spermatozoa cannot recognize the egg; only after these cells have undergone a complex process of maturation in the female tract, known as capacitation, do they express any affinity for the ZP [6].

The constituents of the ZP that mediate sperm–egg recognition are uncertain, with models based on ZP2 or ZP3/4 currently under consideration [7]. Similarly, the identity of the ZP receptors on the surface of mammalian spermatozoa have remained elusive. A variety of candidates have been described in different publications including α-D-mannosidase, arylsulfatase A, β-1,4-galactosyltransferase, fertilization antigen 1, zona pellucida 3 receptor (ZP3R), glutathione–S-transferase, milk fat globule-EGF factor 8, proacrosin, sperm adhesion molecule 1 (SPAM1), Spermadhesins (AWN; AQN-1; AQN-3) and the ZP binding proteins ZPBP1 and ZPBP2 [8], [9]. However gene deletion studies have failed to confirm the exclusive significance of any of these molecules in mediating sperm-egg recognition. An alternative concept, originally proposed by Asquith *et al*. [10], suggested that the biological importance of this event is so great that no single molecular entity can take responsibility for mediating sperm binding to the ZP. Rather, sperm-egg recognition is held to be a highly redundant process mediated by the assembly of a complex array of putative ZP receptors that are brought to the cell surface during capacitation under the influence of molecular chaperones including, in the mouse, endoplasmin (HSP90B1) and heat shock protein 60 (HSPD1). More recently, this model has been refined to propose that chaperones mediate the insertion of ZP recognition molecules into detergent resistant membrane domains (lipid rafts) which move forwards during capacitation to ensure the presentation of these receptor complexes on the anterior aspect of the sperm head where ZP contact will be initiated [11], [12].

This model has been largely developed on the basis of studies conducted in the mouse. Whether this model applies to ZP recognition in the human and, if so, where in this cascade of events, the process becomes impeded in cases of male infertility are unknown. In order to address this question we have conducted a label–free comparative proteomic analysis of sperm proteins in a patient whose spermatozoa exhibit an isolated inability to bind to the ZP in the absence of any other detectable defect. The results of this analysis were then used as the basis for a series of experimental studies, which have provided substantive support for our model of sperm-ZP recognition as a chaperone mediated event involving the assembly and presentation of large multimeric ZP recognition complexes on the sperm surface.

**Table 1:** Sperm peptides found to differ between a fertile donor and an infertile patient.

Retention time (min)	Mono-isotopic Mass (m/z)	P-Value	Ratio Fertile/Infertile	Max. Intensity	ObservedMass	Charge	MS/MS Match		Sequence (Coverage)	MascotScore
34.1	1228.62	<0.001	>10	7686	409.20	3	HSPA2	Heat shock-related 70 kDa protein 2 (IPI00007702)	VEIIANDQGNR (1.7%)	39
							HSPA1L	Heat shock-related 70 kDa protein 1 (IPI00939442)	(1.7%)	
							HSPA1A	Heat shock 70 kDa protein 1A/1B (IPI00304925)	(1.7%)	
							HSPA8	Heat shock cognate 71 kDa protein (IPI00003865)	(1.7%)	
							HSPA5	78 kDa glucose-regulated protein (IPI00003362)	(1.7%)	
42.0	948.47	<0.001	6.46	45864	475.25	2	PGK1/2	Phosphoglycerate kinase, 1/2 (IPI00219568)/(IPI00169383)	VDFNVPMK (1.9%)/(1.9%)	58
24.8	896.43	<0.001	5.15	3023	448.72	2	AKAP4	A-kinase anchor protein 4 (IPI00157860)	EFADSISK (5.4%)	44
49.5	1535.79	<0.001	>10	7130	512.60	3	AKAP4	A-kinase anchor protein 4 (IPI00157860)	MDMSNIVLMLIQK (5.4%)	43
43.7	1380.71	0.02947	6.04	31802	690.85	2	AKAP4	A-kinase anchor protein 4 (IPI00157860)	GYSVGGLLQEVMK (5.4%)	66
38.7	1364.65	0.00321	5.34	63692	682.84	2	AKAP4	A-kinase anchor protein 4 (IPI00157860)	QNATDIMEAMLK (5.4%)	63
39.2	1064.59	0.01868	5.28	25232	532.80	2	AKAP3	A-kinase anchor protein 3 (IPI00290854)	NLLSETIFK (1%)	34
47.7	1718.81	<0.001	>10	7016	573.60	3	RAB2A	Ras-related protein Rab-2A (IPI00031169)	DTFNHLTTWLEDAR(6.6%)	38
50.2	2303.1	0.01053	9.79	40223	768.40	3	ACRBP	Acrosin Binding Protein (IPI00168645)	VSGWLQTEFLSFQDGDFPTK+Deamidated (Q) (3.6%)	32
41.0	1310.70	0.00039	-4.33	30493	655.70	2	H4	Histone H4 (IPI00453473)	TVTAMDVVYALK (11.6%)	20

## Materials and Methods

### Ethics Statement

The experiments described in this study were conducted with the human semen samples obtained with informed written consent from a panel of healthy normozoospermic donors and patients suffering from idiopathic infertility associated with an impaired capacity for fertilization. All experiments conducted in this study were specifically approved by the University of Newcastle Human Research and Ethics Committee and the Melbourne IVF Clinical Research Committee.

### Chemicals and Culture Medium

Unless specified, chemical reagents were obtained from Sigma-Aldrich (St. Louis, MO, USA) and were of research grade. Albumin, ammonium persulfate and 3-[(3-Cholamidopropyl)dimethylammonio]-1-propanesulfonate (CHAPS) were obtained from Research Organics (Cleveland, OH, USA); D-glucose, sodium hydrogen carbonate, sodium chloride, potassium chloride, calcium chloride, potassium orthophosphate, and magnesium sulfate were all analytical reagent grade, purchased from Merck (BDH Merck, Kilsyth, VIC, Australia); ultrapure water was obtained from Fluka (Castle Hill, NSW, Australia); chloroform and methanol were purchased from Fronine (Riverstone, NSW, Australia) at the highest purity available. Tris was from ICN Biochemicals (Castle Hill, NSW, Australia), and acrylamide was from Bio-Rad (Castle Hill, NSW, Australia). Trypsin was purchased from Promega (Annandale, NSW, Australia) and Percoll from (Rydalmere, NSW, Australia). Nitrocellulose was supplied by GE Healthcare (Buckinghamshire, UK) while highly pure Coomassie brilliant blue G250 was obtained from Serva (Heidelberg, Germany). Mouse monoclonal antibody and rabbit polyclonal antibody against HSPA2 were from Sigma-Aldrich. A pan-HSP70 mouse monoclonal antibody was secured from Abcam (ab2787) which recognizes several members of HSP70 family including HSP70, HSC70, GRP78 (HSPA5) and HSP72 (HSPA2). Appropriate HRP conjugated secondary antibodies were obtained from Santa Cruz (Santa Cruz, CA, USA).

**Figure 1:**
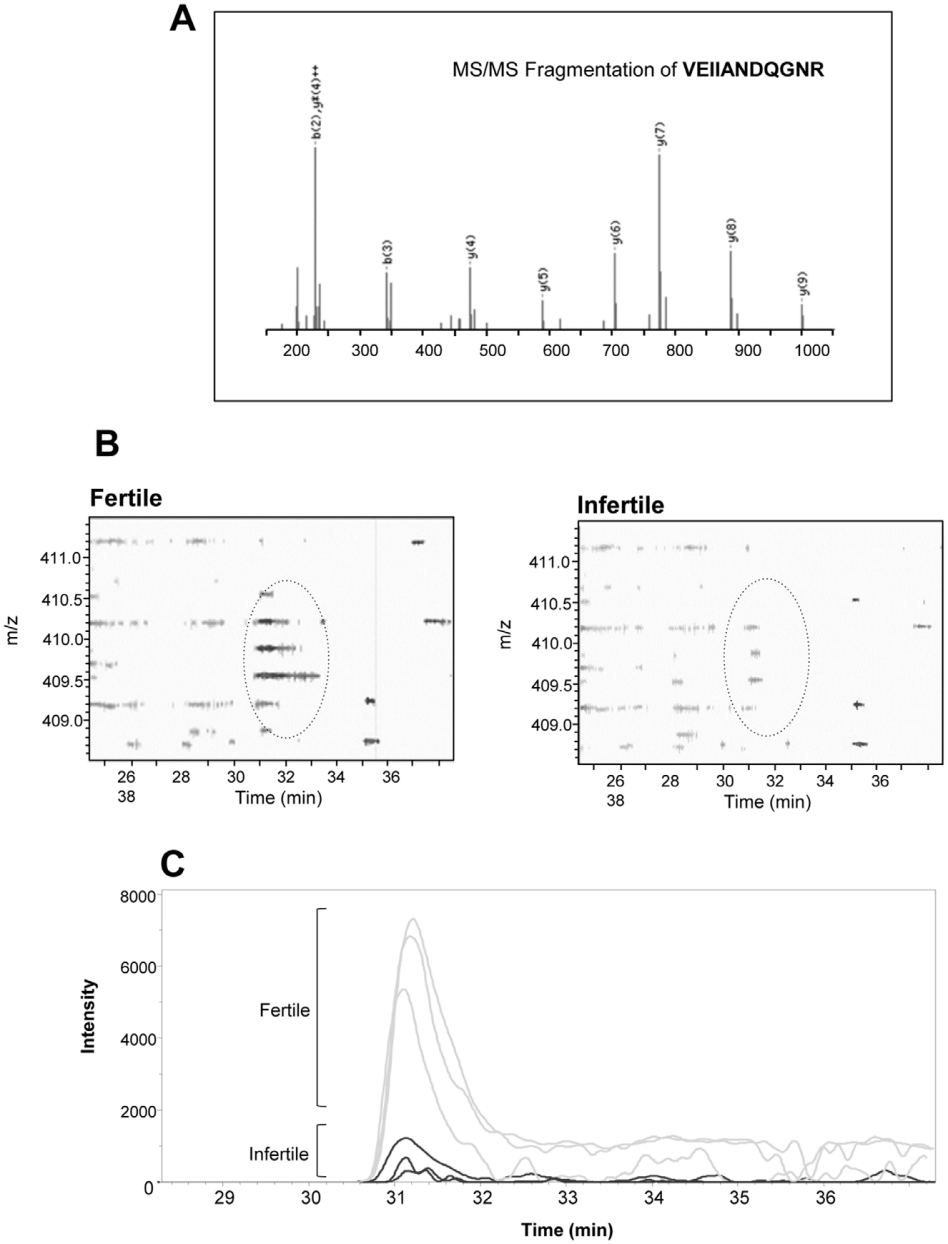
Comparative mass spectrometry analysis of an infertile and a fertile sperm donor. (A) Tandem mass spectrum profile of the monoisotopic ion precursor m/z 409.2 (3^+^) together with the annotated *y* and *b* ion series. The tryptic peptide sequence obtained, VEIIANDQGNR. matched to the protein HSP70 (B) Comparison of a small region of the survey scans generated during the MS analysis of sperm peptides isolated from a fertile donor (left panel) and an infertile patient whose spermatozoa were not capable of recognizing the ZP (right panel). The region of the scan covering the HSP70 peptide is encircled demonstrating the relative lack of this peptide cluster from the sperm proteome of the infertile patient. (C) Extracted ion chromatograms demonstrating the significant underrepresentation of the above HSPA2 peptide in the spermatozoa of the infertile patient compared with a control fertile donor. This proteomic analysis was replicated 3× on each sample.

**Figure 2:**
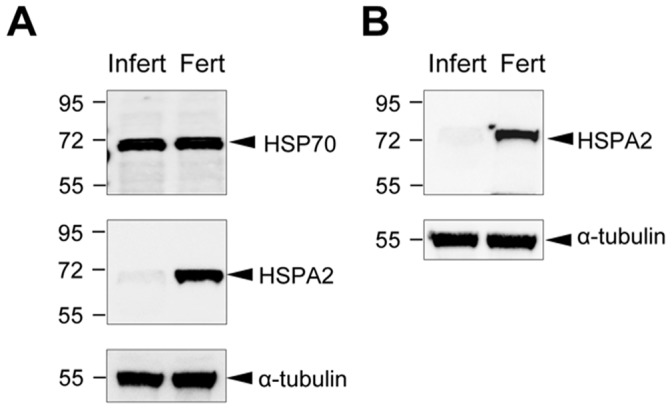
HSPA2 is under-represented in human spermatozoa exhibiting a failure of sperm-ZP binding. (A) Spermatozoa from a patient exhibiting a failure of sperm-ZP interaction were extracted, subjected to SDS PAGE and immunoblotted with antibodies against either HSP70 or HSPA2. The blots were then stripped and reprobed with anti-α-tubulin antibodies to ensure equivalent protein loading in each lane. Similar protein extracts were also prepared from spermatozoa obtained from a fertile donor. These data reveal the selective depletion of HSPA2 from the patient’s spermatozoa. (B) Western blot analysis of spermatozoa from a third patient exhibiting a defect in sperm-egg interaction confirmed the absence of HSPA2 from these cells but not a separate fertile control donor.

### Preparation of Human Spermatozoa

The experiments described in this study were conducted with human semen samples obtained from a panel of healthy normozoospermic donors and patients suffering from idiopathic infertility associated with an impaired capacity for fertilization, in accordance with the University of Newcastle’s Human Ethics Committee guidelines. These samples were collected via masturbation into sterile specimen containers after an abstinence period of 48 h and delivered to the laboratory within 1 h of ejaculation. Purification of human spermatozoa was achieved using 45% and 90% discontinuous Percoll gradients as described previously [11]. Purified spermatozoa were recovered from the base of the 90% Percoll fraction and resuspended in Biggers, Whitten and Whittingham medium (BWW) [13] supplemented with 1 mg/ml PVA (osmolarity of 300 mOsm/kg). The cells were then pelleted by centrifugation at 500 *g* for a further 15 min and finally resuspended at a concentration of 6×10^6^ cells/ml.

### Capacitation of Human Spermatozoa

Capacitation was induced by incubating spermatozoa at 37°C under an atmosphere of 5% CO_2_∶95% air in BWW without CaCl_2_ (BWW-Ca^2+^) but supplemented with 3 mM pentoxifylline and 5 mM dibutyryl cyclic adenosine monophosphate, as described [14]. Incubations were conducted for a period of 3 h, after which the percentage of motile cells was assessed and the spermatozoa were prepared for the various treatments outlined below. Non-capacitated cells were incubated for the same period of time in BWW prepared without NaHCO_3_ (BWW-HCO_3_^-^).

### Immunolocalization of Proteins on Fixed Spermatozoa

Following incubation, spermatozoa were fixed in 4% paraformaldehyde, washed 3× with 0.05 M glycine in phosphate-buffered saline (PBS), plated onto poly-L-lysine coated glass slides and air-dried. All subsequent incubations were performed in a humid chamber at 37°C. The cells were blocked with 10% serum/3% BSA for 1 h, then washed 3× with PBS for 5 min prior to overnight incubation with primary antibody (diluted 1∶100) at 4°C. Slides were then subjected to 3×5 min washes with PBS and incubated with an FITC-conjugated secondary antibody (diluted 1∶500) for 1 h at 37°C. Slides were again washed and mounted in 10% mowiol 4–88 (Calbiochem) with 30% glycerol in 0.2 M Tris (pH 8.5) with 2.5% 1,4-diazobicyclo-(2.2.2)-octane. Cells were finally examined using either a Zeiss Axioplan 2 fluorescence microscope or a Zeiss LSM510 laser scanning confocal microscope equipped with argon and helium/neon lasers (Carl Zeiss, Thornwood, NY, USA).

### Immunodetection of Surface Protein Expression by Flow Cytometry

Capacitated and non-capacitated sperm suspensions were incubated with primary antibody (diluted 1∶100) for 1 h. The cells were subsequently washed 2× with BWW and incubated with FITC-conjugated secondary antibody (diluted 1∶500) for a further 30 min. Following an additional 3 washes with BWW, the cells were incubated with propidium iodide (20 µg/ml) and analysed using a FACSCalibur™ flow cytometer (Becton Dickinson, Franklin Lakes, NJ, USA) with a FL4 530/30 nm band-pass filter, allowing the collection of fluorescence data in logarithmic mode and light-scatter data in linear mode. Ten thousand cells were counted in each sample at a rate of 50–500 events per sec. Data were analyzed using the Cell Quest™ package (Becton Dickinson).

### Duolink Proximity Ligation Assay (PLA)

Duolink *in situ* primary ligation assays (PLA) were conducted in accordance with the manufacturers’ instructions (OLINK Biosciences, Uppsala, Sweden). Briefly, human spermatozoa were purified and capacitated as previously described, after which time they were fixed in 2% paraformaldehyde and coated onto poly-L-lysine slides overnight at 4°C. These cells were then incubated in blocking solution (OLINK Biosciences) for 1 h at 37°C in a humidified chamber, before target proteins were sequentially labeled with a pair of appropriate primary antibodies raised in different species (anti-SPAM1 and anti-ARSA; or anti-ARSA and anti-HSPA2; or anti-SPAM1 and anti-HSPA2; or anti-ARSA and anti-tubulin; or anti-ARSA alone for a negative control) overnight at 4°C in a humidified chamber. After washing, appropriate secondary antibodies (anti-mouse for SPAM1 and tubulin and anti-rabbit for ARSA and HSPA2) conjugated to complementary synthetic oligonucleotides (PLA probes, OLINK Biosciences) were then applied to the samples for 1 h at 37°C. The samples were then sequentially hybridized (15 min), washed and enzymatically ligated (15 min). If the target proteins reside in close proximity, this reaction leads to the production of a signal that appears as a discrete fluorescent dot. These signals were visualized with an Axio Imager A1 fluorescence microscope (Carl Zeiss Microimaging, Inc, Thornwood, NY, USA) and pictures taken using an Olympus DP70 microscope camera (Olympus America, Center Valley, PA, USA).

**Figure 3:**
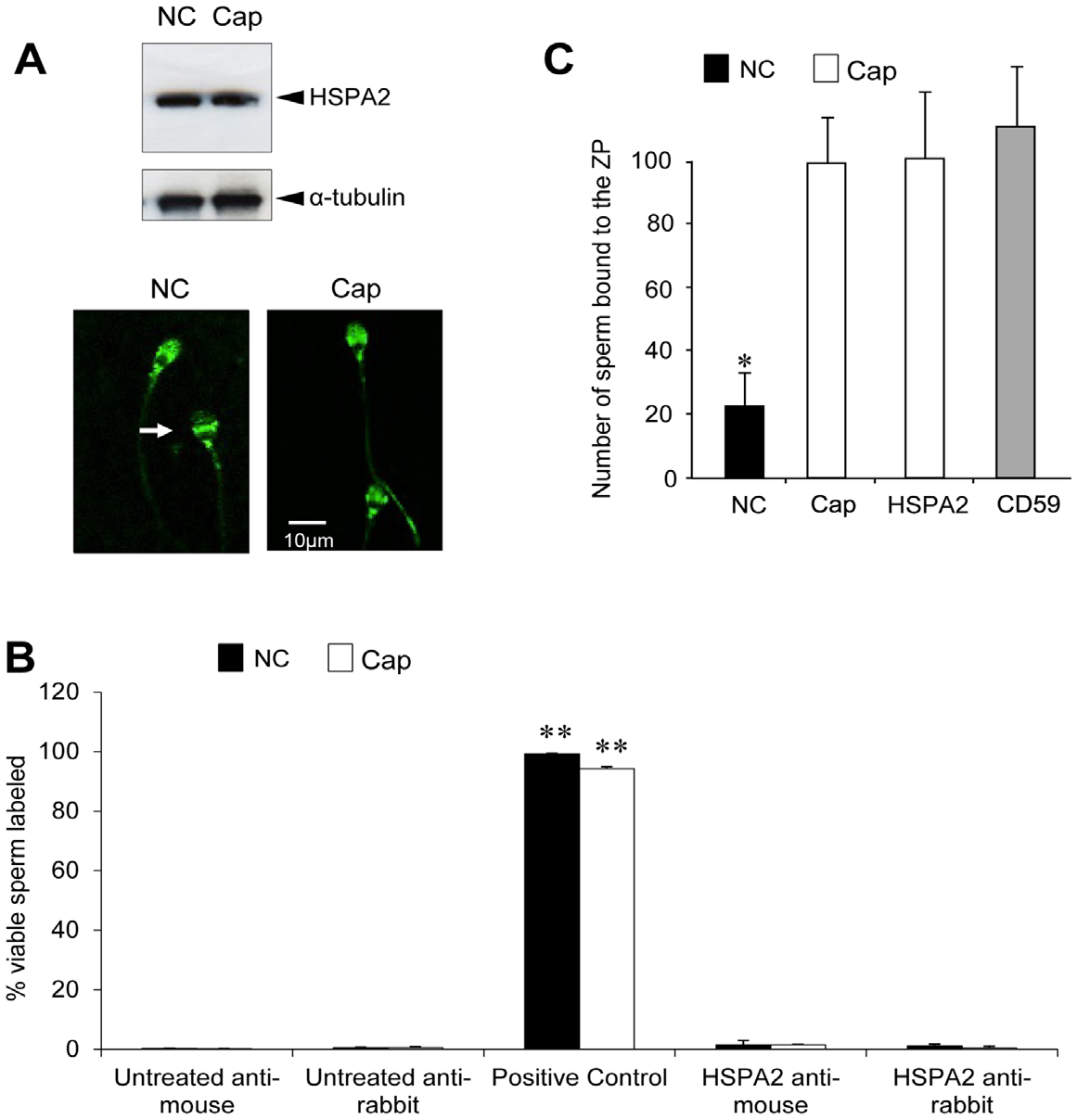
Examination of the role of HSPA2 in ZP adhesion. (A) No change in the relative levels of HSPA2 expression or the localization of the protein could be detected when non-capacitated (NC) and capacitated (Cap) spermatozoa from fertile donors were subjected to either Western blotting or immunocytochemical analysis using anti-HSPA2 monoclonal antibodies. The staining was largely confined to the acrosomal region of the sperm head, with the signal occasionally being more intense in the equatorial segment (arrowed). (B) The use of flow cytometry to examine the distribution of HSPA2 during capacitation revealed a lack of surface expression in both capacitated (Cap) and non-capacitated (NC) spermatozoa regardless of whether a rabbit polyclonal or mouse monoclonal antibody was used for detection purposes. By contrast almost 100% of the cells were labelled with an antibody against the positive control protein, CD59, which is constitutively expressed on the sperm surface. This experiment was repeated 3× with a minimum of 10,000 viable cells scored for each treatment. (C) Preincubation of spermatozoa in the presence of either an anti-HSPA2 or an anti-CD59 monoclonal antibody had no effect on their subsequent ability to bind to the ZP; only the suppression of capacitation by incubating the spermatozoa in bicarbonate-free medium (NC) could significantly influence this process (*P<0.05 compared with all other treatments). All data are presented as mean values ± S.E. Each experiment was replicated 3× on spermatozoa from independent fertile donors.

**Figure 4:**
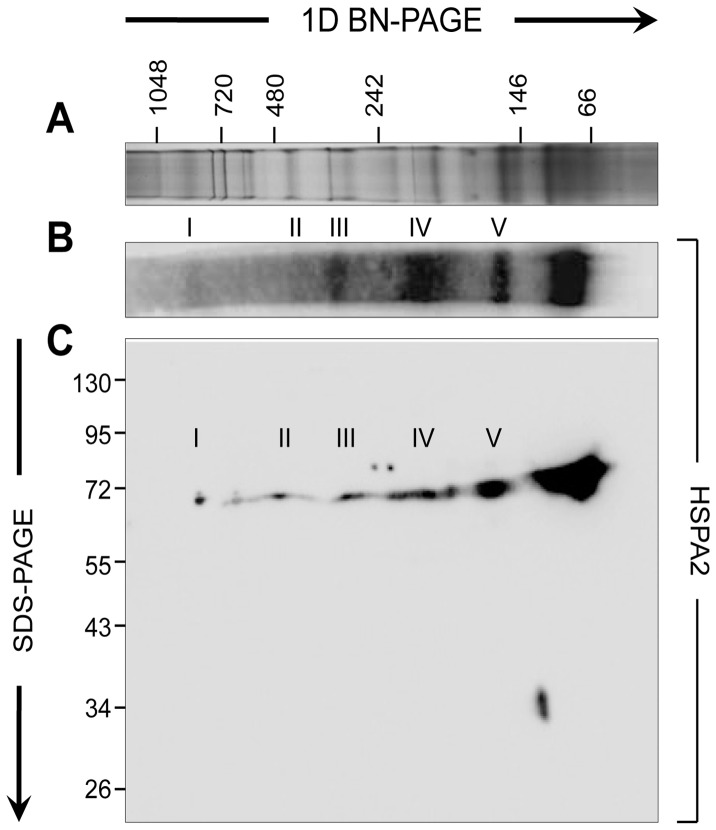
Examination of HSPA2 protein interactions. (A) BN-PAGE was employed to resolve native protein complexes from human spermatozoa. For this purpose, populations of capacitated cells were solubilized in blue native lysis buffer and the extracted proteins were separated on precast 4-16% BN-PAGE gels. (B) BN-PAGE gels were then prepared for immunoblotting with anti-HSPA2. This approach revealed the presence of HSPA2 in six predominant bands, five of which (I – V) resolved at molecular weights beyond that expected for monomeric HSPA2 (∼70 kDa). (C) To confirm the specificity of HSPA2 labelling, 2D BN-PAGE blots were prepared by excising a single lane of a BN-PAGE gel and embedding it at the top of a standard 10% SDS-PAGE gel. Individual proteins were then resolved and immunoblotted with anti-HSPA2 antibodies. Vertically aligned spots indicate the position of HSPA2 within each protein complex, with the larger complexes being located on the left hand side of the image. This experiment was replicated a minimum of 3× and representative gels and immunoblots are shown.

### Blue Native Polyacrylamide Gel Electrophoresis

Following incubation under either capacitating or non-capacitating conditions, suspensions of 1×10^6^ sperm/ml were gently pelleted (300 *g* for 5 min) and resuspended in native protein lysis buffer consisting of 1% n-dodecyl β-D-maltoside which was adjusted to a final concentration below that of the critical micelle concentration, 0.5% Coomassie Blue G250 and a cocktail of protease inhibitors (Roche, Mannheim, Germany). The samples were gently mixed and then incubated at 4°C on an orbital rotator for 30 min. Following incubation, the lysate was recovered by centrifugation at 14,000 *g* for 20 min at 4°C and dialyzed against the Blue native cathode buffer (Invitrogen, Carlsbad, CA), to remove any excess salts and detergents.

For the purpose of one dimensional blue native page (1D BN-PAGE), native protein lysates were loaded onto pre-cast blue native polyacrylamide gels (NativePAGE Novex 4–16%, Bis-Tris; Invitrogen) and resolved using a NativePAGE cathode and anode buffer system (Invitrogen). This system is based in the BN-PAGE technique originally developed by Schagger and von Jagow [15] and optimized for use in spermatozoa by Dun et al., [16] and Redgrove et al., [17], where it has been shown to reproducibly resolve large membrane-protein complexes that retain their native conformation and biological activity. The BN-PAGE electrophoresis apparatus was maintained at 4°C and the samples separated at 100 V until the Coomassie dye front reached the bottom of the loading wells. The voltage was then increased to 200 V and the separation continued until the Coomassie dye front reached the bottom of the gel. The gels were subsequently removed from the electrophoresis apparatus and stained with Coomassie G250. Alternatively, the gels were prepared for either Western blotting or two-dimensional BN-PAGE (2D BN-PAGE). A 200 kDa complex of interest was also carefully excised following BN-PAGE and its proteomic composition determined using an electrospray ionization mass spectrometry interface as previously described.

2D BN-PAGE was conducted in order to resolve native protein complexes into their individual components. For this purpose, individual lanes of the 1D BN-PAGE gel were excised then pre-equilibrated in SDS-PAGE sample buffer (2% w/v SDS, 10% w/v sucrose in 0.1875 M Tris, pH 6.8) supplemented with 0.5% w/v 20 mM dithiothreitol (DTT) and 4% w/v 50 mM iodoacetamide for 10 min. The lanes were then loaded onto a 10% SDS-PAGE gel prepared without stacking wells and sealed in position using 0.5% molten agarose. The gel was placed in a small-format electrophoresis chamber (Bio-Rad Laboratories, Hercules, CA), immersed in SDS-PAGE running buffer and electrophoresed at 100 V until the Coomassie dye front reached the bottom of the gel. Gels were then removed from their cassette and prepared for Western blotting.

### Western Blotting Procedure

Proteins resolved by either SDS-PAGE or BN-PAGE were transferred onto nitrocellulose membranes using conventional Western blotting techniques. In order to detect proteins of interest, membranes were blocked with 3% w/v BSA in Tris-buffered saline (TBS; pH 7.4) supplemented with 0.1% polyoxyethylenesorbitan monolaurate (Tween-20). Membranes were then rinsed in TBS and probed with primary antibody (diluted 1∶1000 in TBS supplemented with 1% BSA and 0.1% Tween-20) for 2 h at room temperature. Following incubation, membranes were washed 3× in TBS containing 0.1% Tween-20 (TBST) for 10 min. Membranes were then probed for 1 h with HRP-conjugated secondary antibody (diluted between 1∶3000–1∶5000 in TBST/1% BSA) at room temperature. Following a further three washes in TBST, cross-reactive proteins were visualized using an enhanced chemiluminescence kit (GE Healthcare) according to the manufacturer’s instructions.

### Human Sperm-zona Pellucida Binding Assay

Capacitated and non-capacitated populations of human spermatozoa were deposited under water-saturated mineral oil at 37°C and 10–20 salt-stored human ova, were added to each sperm suspension and incubated for a further 30 min at 37°C in an atmosphere of 5% CO_2_/95% air. Following co-incubation, the oocytes were washed 3× by serial aspiration through a fine bore glass micropipette to remove any unbound or loosely adhered sperm. The oocytes were then incubated for 10 min in a droplet of BWW supplemented with 5 µg/ml of the DNA-specific fluorochrome 4′,6-diamidino-2-phenylindole (DAPI). The oocytes were then washed in BWW and mounted on glass slides under coverslips supported on pillars comprising 80% paraffin wax and 20% vaseline gel. The number of sperm bound to each ZP was subsequently counted using both phase contrast and fluorescence microscopy (Zeiss Axioplan 2).

### Label Free Comparative Proteomics

The proteomic analysis was based on a label free comparison of 3 independent ejaculates produced by a carefully selected patient attending the Royal Women’s Hospital IVF program due to idiopathic infertility. A careful screen of functional competencies revealed that this normozoospermic individual produced gametes that possessed an isolated inability to bind to the ZP despite exhibiting normal motility and morphology [4]. These samples were compared with 3 independent ejaculates from a normozoospermic fertile donor whose sperm readily bound to the ZP. Spermatozoa were isolated from the samples produced by these individuals following discontinuous Percoll gradient centrifugation as described above and the purified, high density sperm population was recovered and resuspended in medium BWW at a concentration of 50 × 10^6^/ml. These cells were subsequently lysed for 1 h at 4°C in 7 M urea, 2 M thiourea and 4% CHAPS and then centrifuged at 16,000 *g* for 15 min. The supernatant was recovered and sperm proteins were precipitated using a modified methanol:chloroform procedure. Briefly, 2 volumes of methanol were added to an equal volume of cell lysate together with 1 volume of chloroform. The suspensions were then rigorously vortexed and centrifuged (10,000 *g* for 2 min). The upper phase was carefully removed so as to not disturb the interphase, following which 1 vol of methanol were added. The sample was gently inverted 3 times and centrifuged (10,000 *g* for 20 min) after which the supernatant was removed and the pellet left to dry for 5 min per µl. A 200 µg aliquot of this sample was then trypsinized by adding 4 µg of trypsin in 25 mM ammonium bicarbonate and 1 M urea followed by overnight incubation at 37°C with constant agitation. Once this digest had been centrifuged (16,000 *g* for 15 min), the supernatant was transferred to a glass vial and acidified with 0.1% formic acid (FA).

The tryptic peptides were then separated on a Nano-Acquity High Performance Liquid Chromatography system (Waters, Castle Hill, NSW Australia; UHPLC). The UHPLC experiments were performed using the “trapping” set up under the following conditions: Nano-column, C18, 75 µM ID×150 mm, 1.7 mM bead; mobile phase A: 100% water +0.1% FA; mobile phase B: 100% acetonitrile (ACN) +0.1% FA; flow rate trapping, 5 µl min (3 min, 2% B), nano-column, 400 nl/min; gradient, 2–40% B over 40 min, 95% B for 10 min, 2% B for 20 min; loopsize 2 µl and injection volume 1 µl. The column temperature was set to 35°C while the sample temperature was held at 10°C. The LC was connected to a Quadrapole-Time-of-Flight instrument (Micro Q-ToFII; Bruker Daltonic, Preston, VIC, Australia) and precursor masses were recorded at 1 Hz from 50–2000 m/z. The absolute threshold for saving a spectrum was set to 10, while the peak summation width was set to 3. After label-free analysis was performed (see below), the sample was re-injected and the masses, together with the elution time, were used to specifically target only those peptides that were different between infertile and fertile patients for MS/MS analysis.

**Figure 5:**
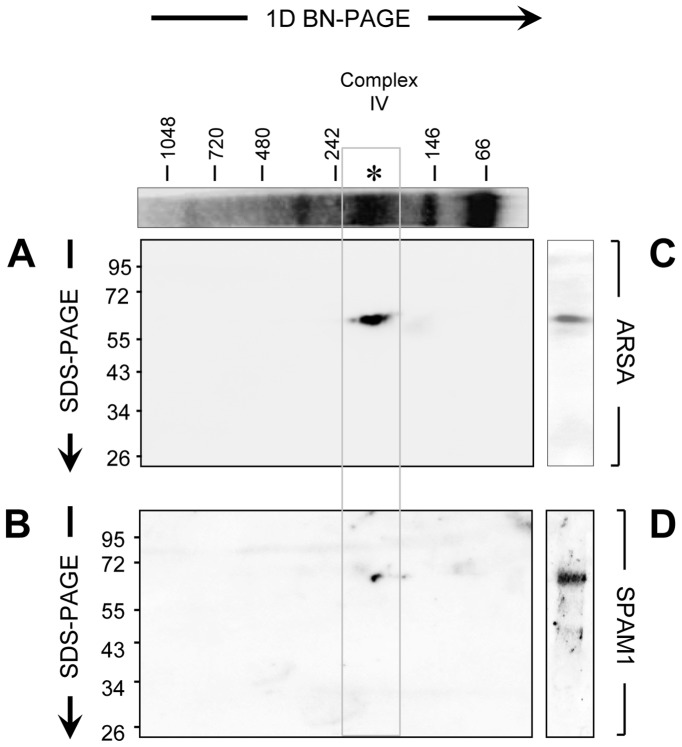
Validation of the proteomic composition of Complex IV. Native protein complexes were extracted from capacitated human spermatozoa and resolved by BN-PAGE in the first dimension and then SDS-PAGE in the second. These 2D BN-PAGE gels were then stained with (A) anti-ARSA antibodies and (B) anti-SPAM1 antibodies (the two proteins selected on the basis of LC-MS/MS sequence analysis of Complex IV) and the blots aligned with the 1D-BN-PAGE stained to reveal the position of the major HSPA2–containing complexes. The presence of ARSA and SPAM1 aligned with the major HSPA2 complex (IV) as indicated with an asterisk. Antibody specificity was also validated through the use of standard SDS-PAGE immunoblots of CHAPS extracted human sperm proteins (C,D), which aligned perfectly with the immunoblots generated by 2D BN-PAGE. Each of these experiments was repeated 3× and representative gels and immunoblots are shown.

**Figure 6:**
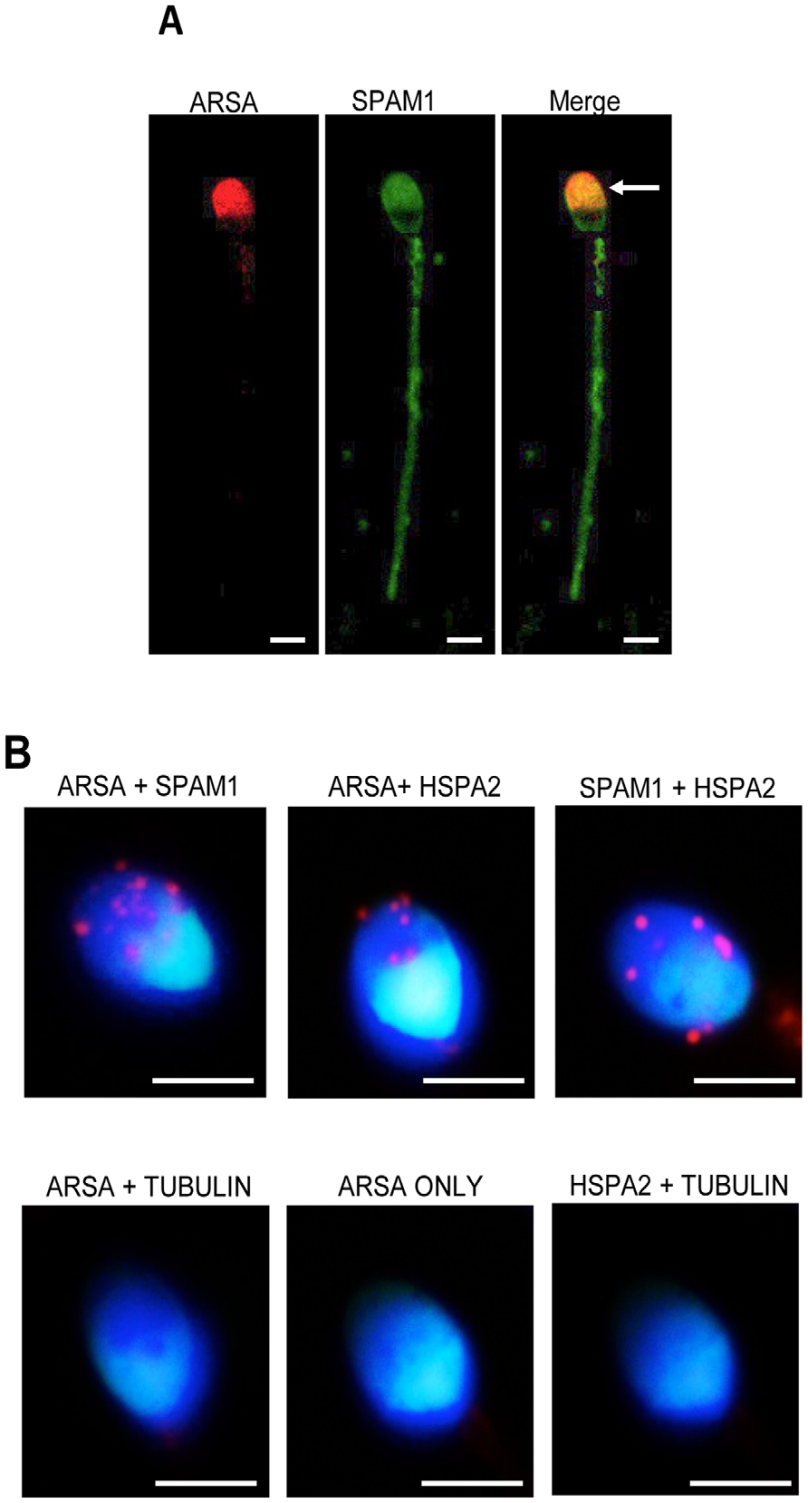
Co-localization of SPAM1 and ARSA in human spermatozoa. (A) Capacitated human spermatozoa were fixed in 2% paraformaldehyde and allowed to settle onto poly-L-lysine slides. These samples were labelled with either anti-ARSA or anti-SPAM1 antibodies followed by either TRITC- or FITC-conjugated secondary antibodies, respectively. The slides were then viewed using confocal microscopy. The merged image clearly shows the co-localization of ARSA and SPAM1 in the acrosomal region of the spermatozoa (arrowed). Scale bar = 3 µM. (B) The close association of HSPA2, ARSA and SPAM1 was also confirmed using a Duolink proximity assay. For this purpose, spermatozoa were incubated with primary antibodies (anti-ARSA, anti-SPAM1, or anti-tubulin) overnight at 4°C and then with oligonucleotide conjugated secondary antibodies (PLA probes) for 1 h at 37°C. The PLA probes were then ligated and the signal was amplified according to the manufacturer’s instructions before being viewed using fluorescence microscopy. Where molecules come into close association (<40 nm) a fluorescent signal is generated as a collection of small pink dots. These data reveal close associations between ARSA and SPAM1, ARSA and HSPA2, SPAM1 and HSPA2. Examples of the unlabelled control incubations including ARSA and tubulin, ARSA alone or HSPA2 and tubulin are shown in the lower panels. Scale bars = 3 µm. All experiments replicated 3× on independent samples.

### Profile Analysis

The “dta” files produced on the micro Q-ToFII were uploaded into profile-analysis and assigned as either “fertile” or “infertile”. To detect peptides, the following setup was used; molecular features, S/N, 8; correlation coefficient, 0.95; minimum compound length, 30 and smooth width, 15. Additional smoothing and proteomics were checked. The program performs both a normalization and time alignment of the peptides, then matches peptides within and across groups. Since the experiment was performed on a high resolution Q-ToF instrument, an accurate mass could be assigned to each peptide to ensure only one peptide was being examined at a time. The total (integrated) intensity for each peptide was then determined and matched with the same peptide from other samples on the basis of its accurate mass and retention time. After the analysis, t-tests were performed to determine whether the signal intensities of each peptide were significantly different between fertile and infertile samples (*n* = 3). Manual validation of any changes observed was then performed by examining the extracted ion-chromatograms of the individual peptides for each biological replicate.

### MS/MS Analysis

Acquired CID spectra were processed in DataAnalysis 4.0, deconvoluted spectra were further analysed with BioTools 3.2 software and submitted to the Mascot database (Mascot 2.2.04, IPI_Human database, V 3.75, 89,486 sequences, 35,847,668 residues). Both peptide mass and fragment mass tolerance were set +/−10 mDa; enzyme specificity was set to trypsin with 2 missed cleavages considered and the following modifications were used: deamidated (NQ), oxidation (M) and phosphorylation (STY). In order to exclude false positive identifications, peptides with Mascot scores below 25 (which was chosen on the basis of manual evaluation of the MS/MS data of peptides with scores below this number) were rejected, unless part of a peptide pair in which one peptide pair had a score above 30. Finally, a false discovery threshold for protein scores <10% was chosen in addition to the score filters. The identified protein sequences were manually validated in BioTools (Bruker Daltonics, Bremen, Germany) on a residue-by residue basis using the raw data to ensure accurate annotation.

### Statistical Analysis

All experiments were replicated at least 3× with independent samples and data are expressed as mean values ± S.E. Statistical analysis was performed using a two-tailed unpaired Student’s t test with Excel. Differences were considered significant for *p*<0.05.

**Figure 7:**
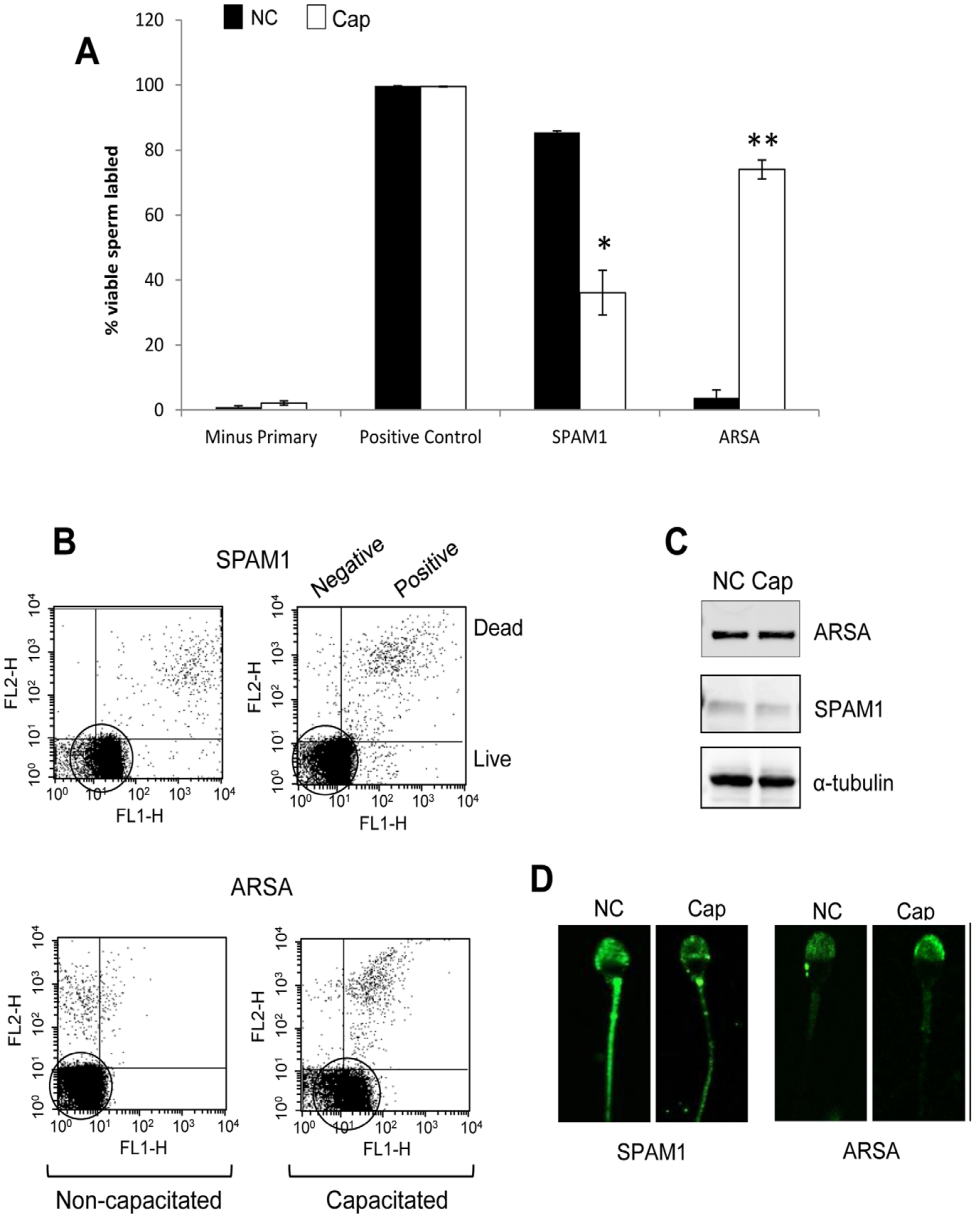
Pattern of SPAM1 and ARSA expression on the surface of live human spermatozoa. Purified human spermatozoa were either held in a non-capacitated state or incubated in capacitating media for 3 h. The presence of SPAM1 and ARSA on the surface of live spermatozoa was assessed using anti-SPAM1 and anti-ARSA primary antibodies in separate aliquots from both treatments (non-capacitated and capacitated), followed by an FITC-conjugated secondary antibody and propidium iodide (PI) as a counterstain to assess cell viability; positive control incubations were labelled with CD59. (A) Graphical representation of percentage of viable cells expressing the target proteins on their surface as detected using FACS. Freshly isolated, non-capacitated cells (solid bars) exhibited SPAM1 surface expression in around 85% of the viable population, while those labelled with ARSA only showed surface expression in less than 5%. Following capacitation however, cells labelled with SPAM1 showed decreased surface expression, such that only 40% of the viable population were labelled after 3 h. In contrast, ARSA surface expression increased during capacitation, resulting in approximately 75% of the viable population being labelled after 3 h. This experiment was repeated 3× with a minimum of 10,000 viable cells scored for each experiment. (B) Representative flow cytometry scattergrams with PI-staining on the Y-axis and FITC-staining on the X-axis. Upper panels: representative of SPAM1 expression, show the majority of viable cells (circled) to be more fluorescent before the induction of sperm capacitation than after. Lower panels: FACS scattergram representative of ARSA staining and showing that the majority of viable cells (circled) increase their fluorescence as the result of capacitation. (C) Western blot analysis showing that capacitation (a process that confers upon spermatozoa the ability to bind to the ZP) was not associated with a change in the total cellular content of ARSA or SPAM1. (D) Similarly, the induction of sperm capacitation was not associated with changes in the subcellular localization of either SPAM1 or ARSA, despite the change in ZP-binding potential. All data are presented as mean values ± S.E. Experiments were replicated 3× on pooled samples (*P<0.05 and **P<0.005 compared with non-capacitated controls).

**Figure 8:**
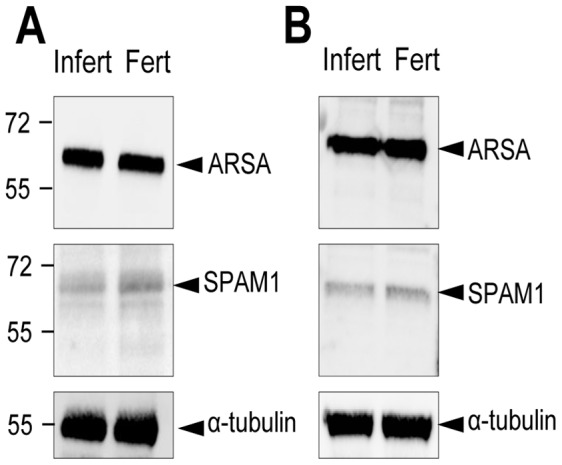
ARSA and SPAM1 are equally represented in human spermatozoa obtained from fertile donors and those exhibiting a failure of sperm-ZP binding. Spermatozoa from two patients (A, B) exhibiting a failure of sperm-ZP interaction were extracted, subjected to SDS PAGE and immunoblotted with antibodies against either ARSA or SPAM1. The blots were then stripped and reprobed with anti-α-tubulin antibodies to ensure equivalent protein loading in each lane. Similar protein extracts were also prepared from spermatozoa obtained from separate fertile donors. These data reveal potential mediators of sperm-egg interaction, SPAM1 and ARSA, are normally expressed in the patients spermatozoa.

## Results and Discussion

### Mass Spectrometry Analysis

Based on the Mascot scores a total of 10 peptides were found to exhibit a change when the proteomic profiles of spermatozoa from a fertile donor were compared with a carefully selected patient exhibiting infertility associated with a very specific defect in sperm-ZP binding (Table 1). This analysis was based on multiple samples from each subject and all of the peptide changes reported were statistically significant (P<0.05). From these peptides, a total of 7 proteins were implicated in this analysis including heat shock 70 kDa protein 2 (HSPA2), the Ras-related protein, Rab-2A, the testes specific isoforms of phosphoglycerate kinase (PGK2), the A-kinase anchor proteins, AKAP3 and AKAP4, acrosin-binding protein (ACRBP) and histone H4 (Table 1). In total, 9 peptides were more highly represented in the fertile spermatozoa compared with the defective specimens that had lost their capacity for binding to the ZP. The only exception to this rule was the histone H4 peptide, which was more highly represented in the infertile patient.

### Significance of Proteomic Changes

These proteomic changes might have reflected the inability of the patient’s spermatozoa to recognize the ZP as a result of two potential mechanisms: (i) they directly reflect the ability of the spermatozoa to undergo the capacitation process whereby ejaculated spermatozoa acquire the ability to recognize the ZP, or (ii) these changes might be indirectly related to sperm-egg recognition because they reflect the underlying normality of the spermatogenic process.

### Changes Reflective of Capacitation

One of the major changes (9.79 fold) was detected in ACRP (mass spectrum and extracted ion chromatograms presented in Figure S1). The protein encoded by this gene is similar to the proacrosin binding protein precursor sp32 found in mouse, guinea pig and pig spermatozoa. It is located in the sperm acrosome and is thought to function as a binding protein for proacrosin that facilitates packaging and condensation of the acrosin zymogen in the acrosomal matrix [18]. However, a second functional role for this protein is suggested by the fact that it is known to become tyrosine phosphorylated during capacitation [10], [19]. The particular peptide identified in this analysis did not contain any tyrosine residues, however, it did contain other amino acids such as serine and threonine that might have been post-translationally modified in concert with tyrosine phosphorylation events affecting other regions of this molecule. Interestingly, AKAP3 and AKAP4, both of which were identified in this analysis, are also well known to become heavily tyrosine phosphorylated during capacitation [20], [21] and again may be indicators for the capacitation status of human spermatozoa (mass spectrum and extracted chromatogram in Figures S2, S3, S4, S5, S6). Similarly Rab-2A is a major sperm protein, which we have previously noted to change during the process of capacitation [22], [23]. Thus the significant decrease in a Rab-2A peptide in the infertile patient (Figure S7) may, like the changes in AKAP3/4 and possibly ACRBP, indicate a defect in the capacitation process that is known to be an absolute pre-requisite for sperm-egg recognition.

### Changes Reflective of Impaired Spermatogenesis

The PGK peptide identified in this analysis is represented in both PGK-1, the X-linked somatic form of the enzyme, and PGK-2, an autosomal sperm specific variant of this molecule. Transcription of the PGK-1 gene is suppressed early in spermatogenesis coinciding with X-chromosome inactivation. In order to compensate for this loss of a key glycolytic enzyme, PGK-2 is transcribed from an intronless testicular retrogene to become the major PGK isoform in spermatozoa, with an essential role in the production of functional cells capable of capacitation and productive interaction with the oocyte [24]-[26]. The fact that sperm motility was normal in the patient’s sample, suggests that sufficient PGK-2 remained in the spermatozoa to support an adequate level of glycolysis [26] and/or that any inadequacies in this context had been compensated for by an increase in oxidative phosphorylation. Because PGK-2 is a major marker for spermiogenesis (the process by which round spermatids differentiate into spermatozoa), it is possible that the under-representation of a PGK peptide in the patient’s spermatozoa is an indirect reflection of some fundamental defect in spermiogenesis (Figure S8).

A similar conclusion might be drawn from the over-representation of histone H4 peptide in this analysis (Figure S9). Thus during spermiogenesis, histones are progressively removed from sperm chromatin and replaced by small basic proteins, called protamines, that facilitate the tight packaging of DNA in the differentiating sperm head. It is well known that defective human spermatozoa are protamine deficient and exhibit significant histone retention [27], [28], [29]. Therefore, the superabundance of a histone H4 peptide in the infertile patient may, like PGK-2 deficiency, signal a fundamental defect in spermiogenesis.

While the proteomic changes observed in this study might reflect defects in sperm capacitation (AKAP3, AKAP4, Rab-2A, ACRBP) or spermiogenesis (PGK-2, histone H4), they do not offer a direct explanation for the loss of ZP binding ability. By contrast, the remaining protein identified in this proteomic analysis, HSPA2, did provide an explanation as to why this specific element of sperm function should be lost in infertile patients.

**Figure 9:**
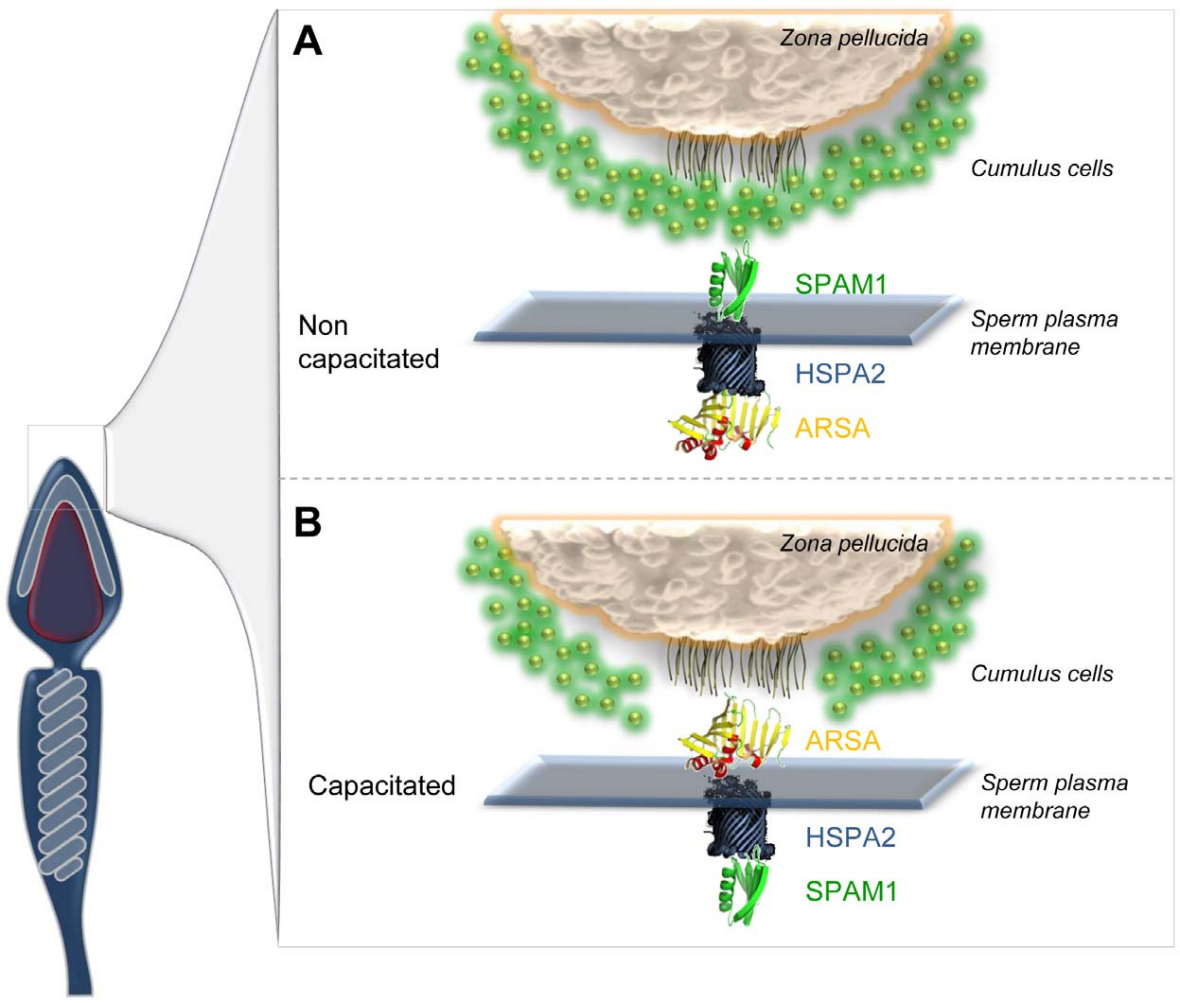
Model for HSPA2 mediated expression of SPAM1 and ARSA on the surface of human spermatozoa. On the basis of our collective findings we propose that HSPA2 coordinates the formation of a multimeric oocyte receptor complex with SPAM1 and ARSA in human spermatozoa. This complex is orientated such that the hyaluronidase, SPAM1, is expressed on the surface of the majority of non-capacitated cells and is thus positioned to mediate binding and dispersion of the hyaluronic rich matrix of the cumulus mass. As these cells penetrate the cumulus mass and complete their capacitation, the receptor complex is reorientated such that the cells now express ARSA, one of the putative receptors for the ZP, on their surface. This reorientation process involves the mediation of HSPA2.

### Evidence that HSPA2 Plays a Key Role in Human Sperm-ZP Adhesion

Our interest in the role of molecular chaperones in mediating sperm–egg recognition stems from earlier studies in the mouse, where such molecules were shown to play a key role in assembling and presenting zona recognition complexes to the sperm surface during capacitation [10]. The present proteomic analysis revealed that a peptide shared by several members of the HSP70 family was significantly (P<0.001) under-represented in multiple ejaculates from the patient exhibiting a failure in sperm-egg recognition compared with a normal donor, as demonstrated by both the survey scans and multiple extracted ion chromatograms presented in Figure 1. This observation was of particular interest because one member of this family, namely HSPA2, has previously been linked with the differentiation of functional spermatozoa [30], [31], [32], [33]. In order to investigate this association further, a second patient was identified with a history of failed fertilization associated with impaired ZP-binding and his spermatozoa were examined for the presence of HSP70-family proteins by Western blot analyses. Using a pan-HSP70 polyclonal antibody that recognized several members of the HSP70 family (including: HSP70, HSC70, HSPA5 and HSPA2), the overall level of HSP70 expression was found to be unchanged in this patient compared with a fertile control donor (Figure 2A). However, when the same blot was stripped and re-probed with a monoclonal antibody that uniquely recognises HSPA2, a testis-specific HSP70 isoform that has previously been correlated with male infertility [30], [32], [33], [34], a clear deficiency in this specific chaperone was observed (Figure 2A). Analysis of a third patient exhibiting the same phenotype, confirmed the lack of HSPA2 expression in defective spermatozoa exhibiting an inability to bind to the ZP (Figure 2B). Thus a striking lack of HSPA2 was a consistent feature of all three patients exhibiting an isolated defect in the capacity of their spermatozoa to interact with the ZP.

### Analysis of the Mechanisms by which HSPA2 Mediates Sperm–egg Recognition

#### Subcellular localization of HSPA2

Initial exploration of the fundamental mechanisms by which HSPA2 regulates sperm–egg interaction indicated that the capacitation-dependent increase in zona binding ability was not associated with a significant change in either the HSPA2 content of human spermatozoa or the localization of this protein at the light microscope level (Figure 3A). Immunocytochemically, this chaperone appeared to be associated with the neck domain, at the junction between the flagellum and the sperm head, and the acrosomal region, occasionally accompanied by a particularly strong signal in the equatorial segment, regardless of the capacitation status of the cells (Figure 3A). Flow cytometric analyses of live cells also revealed that HSPA2 is never expressed on the surface of human spermatozoa. Thus while these cells were readily stained with anti-CD59, a surface-expressed complement regulatory protein, no equivalent signal was generated by spermatozoa labelled with anti-HSPA2 antibody, before or after capacitation (Figure 3B). In keeping with this apparent lack of surface expression, we also confirmed that treatment of live cells with an anti-HSPA2 antibody had no effect on the ability of spermatozoa to adhere to the ZP, regardless of the capacitation status of these cells (Figure 3C). Taken together, these observations suggested that while HSPA2 may play a key role in mediating sperm-egg recognition, this chaperone is unlikely to be directly involved in the binding of human spermatozoa to the ZP surface. However, this conclusion did not rule out the possibility that HSPA2 participates indirectly in fertilization by mediating the assembly and/or presentation of zona recognition complexes on the sperm surface [10], [12], [17], [35]. Consistent with this hypothesis, HSPA2 has been independently found to partition into membrane rafts in human spermatozoa and these membrane microdomains have, in turn, been shown to possess affinity for homologous ZP [12].

#### HSPA2 is part of a functional zona recognition complex

To explore the possible role of HSPA2 in zona receptor complex formation we employed BN-PAGE, a technique that we have successfully used in previous studies to identify chaperone client proteins in both mouse and human spermatozoa [17], [35]. In the present case, application of BN-PAGE successfully resolved in excess of 15 predominant protein complexes in sperm lysates with aggregate molecular weights greater than 150 kDa (Figure 4A).

Labelling of 1D BN-PAGE Western blots with anti-HSPA2 antibodies revealed the presence of this chaperone in a subset of high molecular weight complexes (approximately 150-900 kDa) in addition to a predominant band at approximately 70 kDa, representing the HSPA2 monomer (Figure 4B). The specificity of this labelling was demonstrated though the application of 2D BN-PAGE to resolve each of these complexes into their individual constituents. As anticipated, anti-HSPA2 labelling of 2D BN-PAGE Western blots identified a single protein of approximately 70 kDa within each of the six protein bands originally detected in 1D BN-PAGE blots (Figure 4C).

Collectively, these results supported the concept that HSPA2 is indirectly involved in human sperm-ZP interaction through the assembly of multimeric zona receptor complexes. To provide proof-of-principle for this model, the major HSPA2 labelled complex (Complex IV; Figure 4B) was excised and subjected to proteomic analysis using a 1D nano LC-ESI MS/MS interface (Table S1). This analysis definitively confirmed the presence of HSPA2 in this complex, and also identified two additional proteins, SPAM1 and ARSA, both of which have been implicated in sperm-oocyte interactions. The former possesses hyaluronidase activity and has been linked with dispersal of the cumulus mass, while the latter has been associated with adhesion to the ZP [36], [37], [38], [39]. The combined molecular weight of these three proteins (202 kDa) is similar to that determined experimentally for Complex IV as a whole (approximately 200 kDa), suggesting that they are, in fact, the only major components of the complex. Furthermore, the presence of these proteins in Complex IV was validated by immunoblotting 2D BN-PAGE gels with anti-ARSA and anti-SPAM1 antibodies (Figure 5). In these blots, ARSA was clearly detected as a single spot of the appropriate molecular weight (63 kDa) that vertically aligned with Complex IV (Figure 5A, C) while SPAM1 2D blots revealed a single spot of appropriate molecular weight (67 kDa) that again aligned with Complex IV (Figure 5B, D).

#### Co-localization of HSPA2 with ARSA and SPAM1 in a zona recognition complex

Given the novelty and possible significance of Complex IV in ZP adhesion, we next sought to confirm the co-localization of SPAM1, ARSA and HSPA2 in a single complex using a variety of independent methods. Initially, intracellular localization of ARSA and SPAM1 was examined in fixed but non-permeabilized populations of non-capacitated and capacitated human spermatozoa. Consistent with a proposed role in the mediation of sperm-ZP interaction, both proteins were co-localized in the peri-acrosomal domain of the sperm head, in addition to more modest labelling of the mid- and/or principal-piece of the tail in the case of SPAM1 (Figure 6A). An *in situ* proximity ligation assay (Duolink) was then used to confirm the close association of these proteins in an integrated complex. In this assay, the fluorescent signal produced as a result of the intimate association between HSPA2, ARSA and SPAM1 generated a number of discrete red spots located primarily over the acrosomal region of the sperm head in >85% cells examined (Figure 6B). The specificity of this labelling pattern was confirmed through the use of an irrelevant antibody (anti-α-tubulin), which was an isotype match for anti-ARSA and anti-SPAM1 (Figure 6B).

#### Surface expression of ARSA and SPAM1

The tight articulation of HSPA2, ARSA and SPAM1 is consistent with the notion that these molecules associate into a single recognition complex, which participates in sperm-egg recognition in a capacitation-dependent manner. In order to test this hypothesis we used flow cytometry to monitor the surface expression of ARSA and SPAM1 in spermatozoa before and after capacitation. The results of this assay revealed that the surface expression profiles of both these HSPA2 client proteins was dramatically, yet reciprocally, influenced by the capacitation status of the cells (Figure 7A, B). Thus, in non-capacitated cells approximately 85% of the spermatozoa were surface labelled with SPAM1. However, following the initiation of capacitation, the number of cells that were surface labelled for this molecule decreased significantly with time such that after 180 min it was only observed in approximately 40% of the live cell population (Figure 7A, B). In marked contrast, the surface expression of ARSA followed the opposite trend and increased significantly during capacitation. Thus this protein was only observed on the surface of less than 5% of spermatozoa in the absence of capacitation whereas, following the induction of this process, approximately 75% of cells were positive (Figure 7A, B).

This regulated shift in surface expression from SPAM1 to ARSA perfectly matches the functional requirements of spermatozoa engaged in the process of fertilization. Thus, as spermatozoa approach the oocyte they first encounter the hyaluronic rich matrix of the cumulus mass. At this point the surface expression of SPAM1, a hylauronidase, is required to aid in penetration of the cumulus matrix [37], [38], [40]. As these cells penetrate the cumulus mass and complete their capacitation, they then express ARSA on their surface, one of the putative receptors for the ZP [36], [39], [41].

These changes in surface location were not accompanied by any net loss or gain of protein from these cells as demonstrated by both Western blot analysis and immunocytochemistry (Figure 7C, D). Similarly, the lost fertilizing potential of the patients described in Figure 2, was not associated with a loss of ARSA and SPAM1, both of which were found to be present in the spermatozoa by Western blot analysis (Figure 8). HSPA2 was the only component of the recognition complex that was missing in these patients. In light of these data, we conclude that the functionality of SPAM1 and ARSA is critically dependent on the concomitant presence of HSPA2 to orchestrate the regulated surface expression of these molecules.

### Central Role of HSPA2 in Male Reproduction

#### Spermatogenesis

This study reinforces the notion that HSPA2 plays a major role in male reproduction orchestrating both spermatogenesis and ultimate expression of sperm function. At a testicular level HSPA2 has been shown to play an essential role in the transition of mouse spermatogenic cells through the late meiotic stages of spermatogenesis [41], [42]. Within these cells, HSPA2 supports the formation of a heterodimeric complex between CDC2 and cyclin B1 during the transition between G_1_ to S-phase and then from G_2_ to M-phases of meiosis [43]. In addition, it acts as a component of the synaptonemal complex [41] and assists with chromosome cross-over during meiosis. More recent analyses of post-meiotic germ cells have revealed that, after the completion of meiosis, HSPA2 acquires a new, unexpected function as a chaperone of spermatid-specific DNA packaging transition protein [44]. The latter serve as intermediaries, replacing histones before themselves being replaced by protamines during spermiogenesis. These findings suggest that HSPA2 is a major regulator of chromatin remodelling in differentiating spermatids and, in its absence, abnormal spermatozoa will be produced characterized by poorly compacted chromatin, in keeping with the over-expression of histone H4 observed in this study. It is also apparent that since HSPA2 is of central importance to meiosis, the absence of this chaperone from the spermatozoa of these normozoospermic patients, must represent a post-meiotic defect in the spermatogenic process.

#### Sperm function

The under-representation of peptides from AKAP3/4, Rab-2A and ACRBP, suggest an impairment in the process of sperm capacitation which confers upon spermatozoa the ability to participate in sperm-egg recognition. [8], [9], [10], [35]. Our data suggest that this process is impaired in pathologically defective spermatozoa because of an inadequacy in the presence of a key chaperone, HSPA2 which, in addition to all its other roles, also plays a key role in the assembly and expression of receptors on the sperm surface capable of mediating interaction with the cumulus-oocyte complex [30]. Indeed, the binding of human spermatozoa to hyaluronic acid polymers is currently used as a diagnostic test in selecting high quality spermatozoa for assisted conception therapy [30], [31]. The present results offer a rational explanation as to why HSPA2 expression, hyaluronic acid binding and sperm-zona interaction are functionally linked and why they are all associated with male infertility; without HSPA2, neither the hyaluronidase receptor, SPAM1, nor the zona receptor, ARSA, would be expressed in the co-ordinated manner needed to achieve fertilization (Figure 9).

In view of the fact that HSPA2 was identified as a key constituent of a number of high molecular weight complexes (Fig. 4), some of which have previously been shown to possess zona affinity [17], it is considered unlikely that the complex we have characterized is uniquely responsible for the mediation of human sperm-ZP recognition. Such a conclusion is consistent with the demonstration that male mice bearing targeted deletions of either ARSA [45] or SPAM1 [46] retain their fertility. It will therefore be of considerable interest to investigate the molecular composition of the additional HSPA2 complexes to determine the complete array of proteins involved in regulating sperm–egg recognition during human fertilization.

## Supporting Information

Figure S1 **Acrosin Binding Protein (ACRBP) peptide.** (A) Tandem mass spectra profile of the monoisotopic ion precursor m/z 768.4 (2^+^) together with the annotated *y* and *b* ion series. The tryptic peptide sequence obtained, VSGWLQTEFLSFQDGDFPTK, matched to the protein ACRBP. (B) Extracted ion chromatograms (EIC) from samples collected using MS-only precursor scans (1 Hz, 50–2000 Da). A significant difference (*p* = 0.011) is demonstrated when the EIC from 3 independent semen samples from a fertile donor (green traces) are overlayed with 3 independent samples from an infertile patient (blue traces) whose spermatozoa could not bind to the ZP.
(TIF)

Figure S2 **A-kinase anchoring protein AKAP3.** (A) Tandem mass spectra profile of the monoisotopic ion precursor m/z 532.8 (2^+^) together with the annotated *y* and *b* ion series. The tryptic peptide sequence obtained, NLLSETIFK, matched to the protein AKAP3. (B) Extracted ion chromatograms (EIC) from samples collected using MS-only precursor scans (1 Hz, 50–2000 Da). A significant difference (*p* = 0.019) is demonstrated when the EIC from 3 independent semen samples from a fertile donor (green traces) are overlayed with 3 independent samples from an infertile patient (blue traces) whose spermatozoa could not bind to the ZP.
(TIF)

Figure S3 **A-kinase anchoring protein AKAP4.** (A) Tandem mass spectra profile of the monoisotopic ion precursor m/z 448.72 (2^+^) together with the annotated *y* and *b* ion series. The tryptic peptide sequence obtained, EFADSISK, matched to the protein AKAP4. (B) Extracted ion chromatograms (EIC) from samples collected using MS-only precursor scans (1 Hz, 50–2000 Da). A significant difference (*p*<0.001) is demonstrated when the EIC from 3 independent semen samples from a fertile donor (green traces) are overlayed with 3 independent samples from an infertile patient (blue traces) whose spermatozoa could not bind to the ZP.
(TIF)

Figure S4 **A-kinase anchoring protein AKAP4.** (A) Tandem mass spectra profile of the monoisotopic ion precursor m/z 682.84 (2^+^) together with the annotated *y* and *b* ion series. The tryptic peptide sequence obtained, QNATDIMEAMLK, matched to the protein AKAP4. (B) Extracted ion chromatograms (EIC) from samples collected using MS-only precursor scans (1 Hz, 50–2000 Da). A significant difference (*p* = 0.003) is demonstrated when the EIC from 3 independent semen samples from a fertile donor (green traces) are overlayed with 3 independent samples from an infertile patient (blue traces) whose spermatozoa could not bind to the ZP.
(TIF)

Figure S5 **A-kinase anchoring protein AKAP4.** (A) Tandem mass spectra profile of the monoisotopic ion precursor m/z 690.85 (2^+^) together with the annotated *y* and *b* ion series. The tryptic peptide sequence obtained, GYSVGGLLQEVMK, matched to the protein AKAP4. (B) Extracted ion chromatograms (EIC) from samples collected using MS-only precursor scans (1 Hz, 50–2000 Da). A significant difference (*p* = 0.029) is demonstrated when the EIC from 3 independent semen samples from a fertile donor (green traces) are overlayed with 3 independent samples from an infertile patient (blue traces) whose spermatozoa could not bind to the ZP.
(TIF)

Figure S6 **A-kinase anchoring protein AKAP4.** (A) Tandem mass spectra profile of the monoisotopic ion precursor m/z 512.60 (3^+^) together with the annotated *y* and *b* ion series. The tryptic peptide sequence obtained, MDMSNIVLMLIQK, matched to the protein AKAP4. (B) Extracted ion chromatograms (EIC) from samples collected using MS-only precursor scans (1 Hz, 50–2000 Da). A significant difference (*p*<0.001) is demonstrated when the EIC from 3 independent semen samples from a fertile donor (green traces) are overlayed with 3 independent samples from an infertile patient (blue traces) whose spermatozoa could not bind to the ZP.
(TIF)

Figure S7 **Ras related protein, Rab2A.** (A) Tandem mass spectra profile of the monoisotopic ion precursor m/z 573.60 (3^+^) together with the annotated *y* and *b* ion series. The tryptic peptide sequence obtained, DTFNHLTTWLEDAR, matched to the protein, Ras related protein, Rab2A. (B) Extracted ion chromatograms (EIC) from samples collected using MS-only precursor scans (1 Hz, 50–2000 Da). A significant difference (*p*<0.001) is demonstrated when the EIC from 3 independent semen samples from a fertile donor (green traces) are overlayed with 3 independent samples from an infertile patient (blue traces) whose spermatozoa could not bind to the ZP.
(TIF)

Figure S8 **Phosphoglycerate kinase, PGK-1/2.** (A) Tandem mass spectra profile of the monoisotopic ion precursor m/z 475.25 (3^+^) together with the annotated *y* and *b* ion series. The tryptic peptide sequence obtained, VDFNVPMK, matched to the protein PGK-1/2. (B) Extracted ion chromatograms (EIC) from samples collected using MS-only precursor scans (1 Hz, 50–2000 Da). A significant difference (*p*<0.001) is demonstrated when the EIC from 3 independent semen samples from a fertile donor (green traces) are overlayed with 3 independent samples from an infertile patient (blue traces) whose spermatozoa could not bind to the ZP.
(TIF)

Figure S9 **Histone H4.** (A) Tandem mass spectra profile of the monoisotopic ion precursor m/z 655.70 (2^+^) together with the annotated *y* and *b* ion series. The tryptic peptide sequence obtained, TVTAMDVVYALK, matched to the protein, Histone H4. (B) Extracted ion chromatograms (EIC) from samples collected using MS-only precursor scans (1 Hz, 50–2000 Da). A significant difference (*p*<0.001) is demonstrated when the EIC from 3 independent semen samples from a fertile donor (green traces) are overlayed with 3 independent samples from an infertile patient (blue traces) whose spermatozoa could not bind to the ZP.
(TIF)

Table S1 **Protein identities of 200 kDa complex obtained via MS/MS analysis.**
(DOC)
