# Supplementary figures and images for: The Molecular Chaperone HSPA2 Plays a Key Role in Regulating the Expression of Sperm Surface Receptors That Mediate Sperm-Egg Recognition
Source: PLoS One. 2012 Nov 29;7(11):e50851. doi: 10.1371/journal.pone.0050851 (PMC3510172; doi:10.1371/journal.pone.0050851)

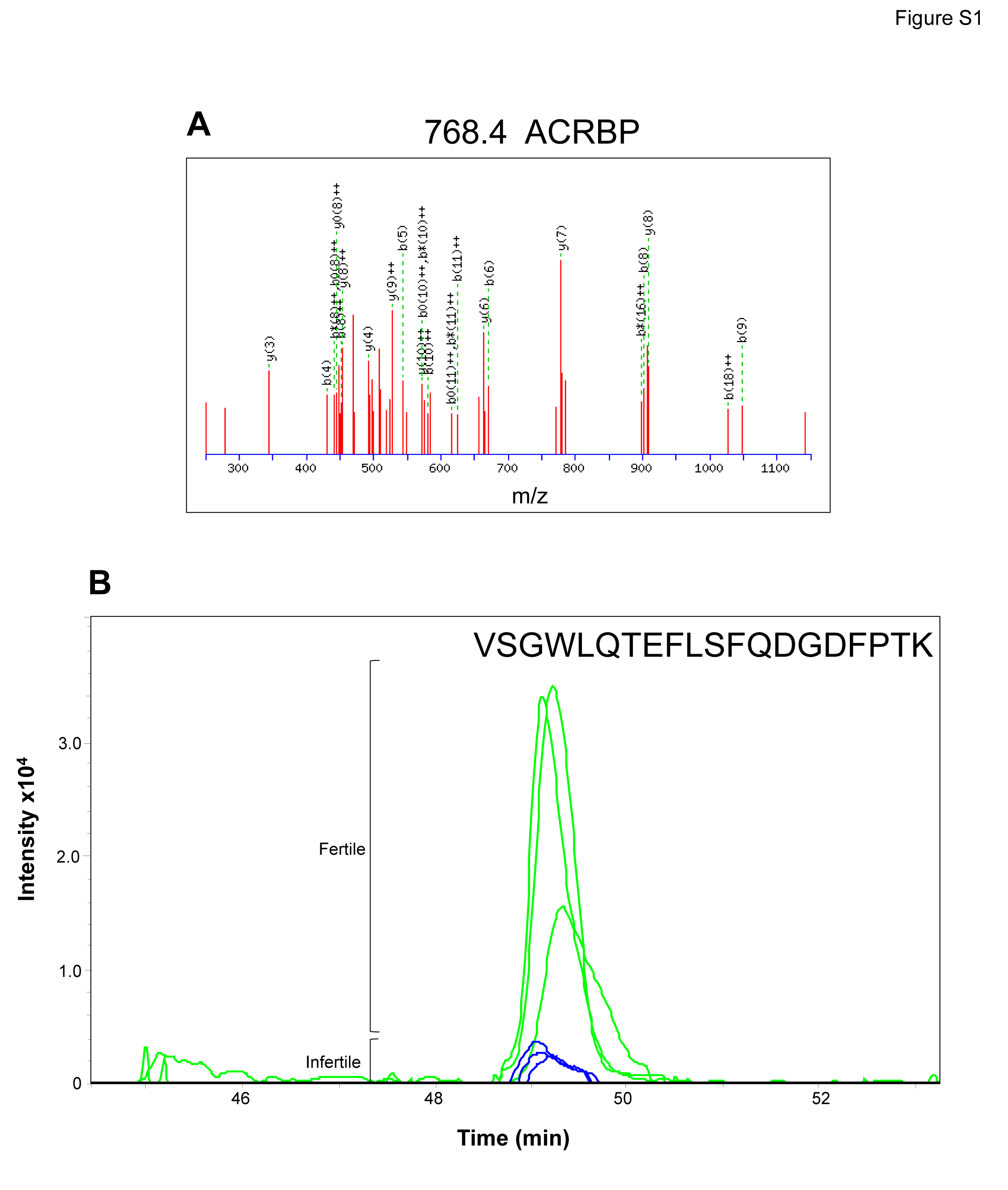

Supplement: Figure S1 — Acrosin Binding Protein (ACRBP) peptide. (A) Tandem mass spectra profile of the monoisotopic ion precursor m/z 768.4 (2+) together with the annotated y and b ion series. The tryptic peptide sequence obtained, VSGWLQTEFLSFQDGDFPTK, matched to the protein ACRBP. (B) Extracted ion chromatograms (EIC) from samples collected using MS-only precursor scans (1 Hz, 50–2000 Da). A significant difference (p = 0.011) is demonstrated when the EIC from 3 independent semen samples from a fertile donor (green traces) are overlayed with 3 independent samples from an infertile patient (blue traces) whose spermatozoa could not bind to the ZP. (TIF) [file pone.0050851.s001.tif]

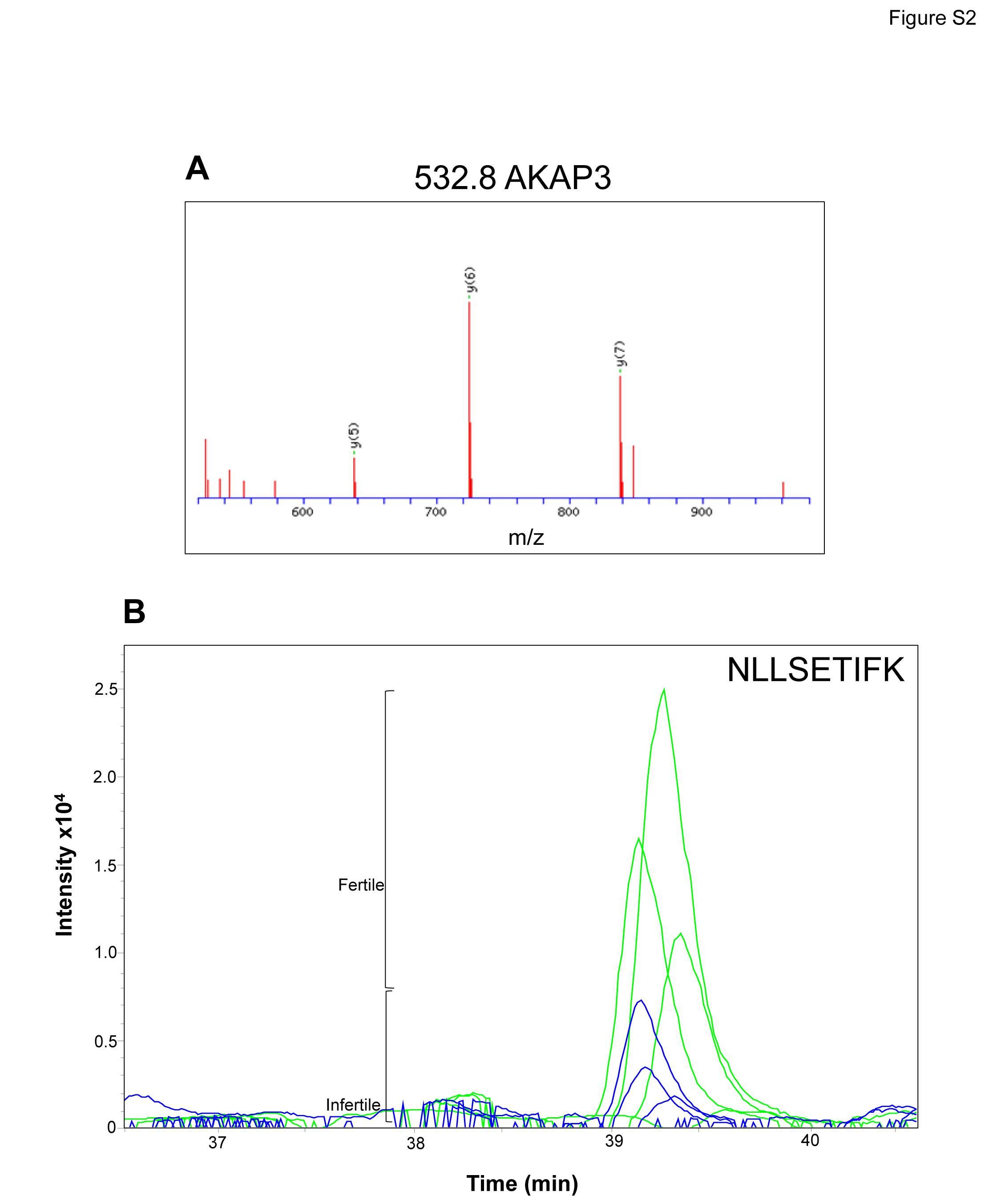

Supplement: Figure S2 — A-kinase anchoring protein AKAP3. (A) Tandem mass spectra profile of the monoisotopic ion precursor m/z 532.8 (2+) together with the annotated y and b ion series. The tryptic peptide sequence obtained, NLLSETIFK, matched to the protein AKAP3. (B) Extracted ion chromatograms (EIC) from samples collected using MS-only precursor scans (1 Hz, 50–2000 Da). A significant difference (p = 0.019) is demonstrated when the EIC from 3 independent semen samples from a fertile donor (green traces) are overlayed with 3 independent samples from an infertile patient (blue traces) whose spermatozoa could not bind to the ZP. (TIF) [file pone.0050851.s002.tif]

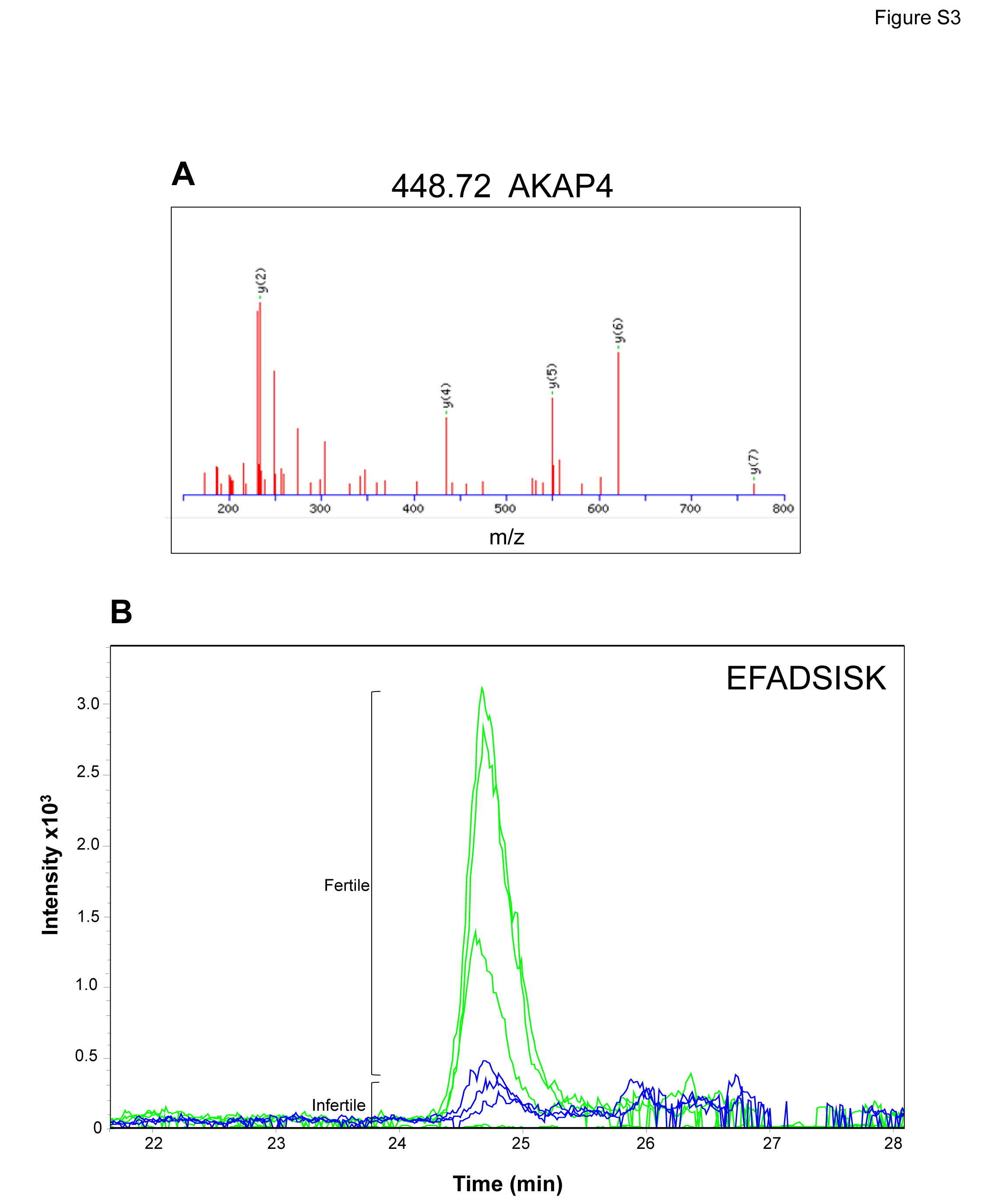

Supplement: Figure S3 — A-kinase anchoring protein AKAP4. (A) Tandem mass spectra profile of the monoisotopic ion precursor m/z 448.72 (2+) together with the annotated y and b ion series. The tryptic peptide sequence obtained, EFADSISK, matched to the protein AKAP4. (B) Extracted ion chromatograms (EIC) from samples collected using MS-only precursor scans (1 Hz, 50–2000 Da). A significant difference (p<0.001) is demonstrated when the EIC from 3 independent semen samples from a fertile donor (green traces) are overlayed with 3 independent samples from an infertile patient (blue traces) whose spermatozoa could not bind to the ZP. (TIF) [file pone.0050851.s003.tif]

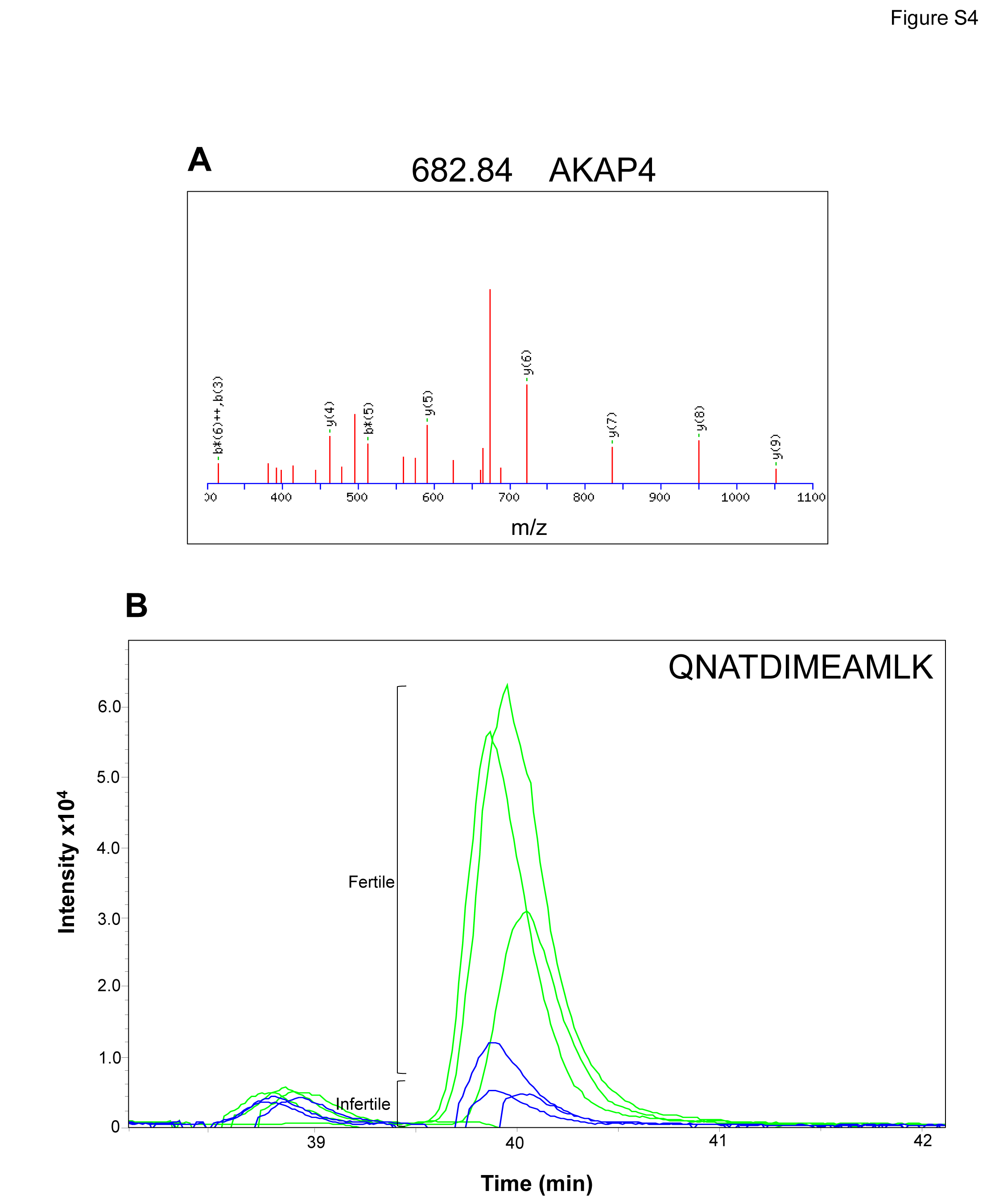

Supplement: Figure S4 — A-kinase anchoring protein AKAP4. (A) Tandem mass spectra profile of the monoisotopic ion precursor m/z 682.84 (2+) together with the annotated y and b ion series. The tryptic peptide sequence obtained, QNATDIMEAMLK, matched to the protein AKAP4. (B) Extracted ion chromatograms (EIC) from samples collected using MS-only precursor scans (1 Hz, 50–2000 Da). A significant difference (p = 0.003) is demonstrated when the EIC from 3 independent semen samples from a fertile donor (green traces) are overlayed with 3 independent samples from an infertile patient (blue traces) whose spermatozoa could not bind to the ZP. (TIF) [file pone.0050851.s004.tif]

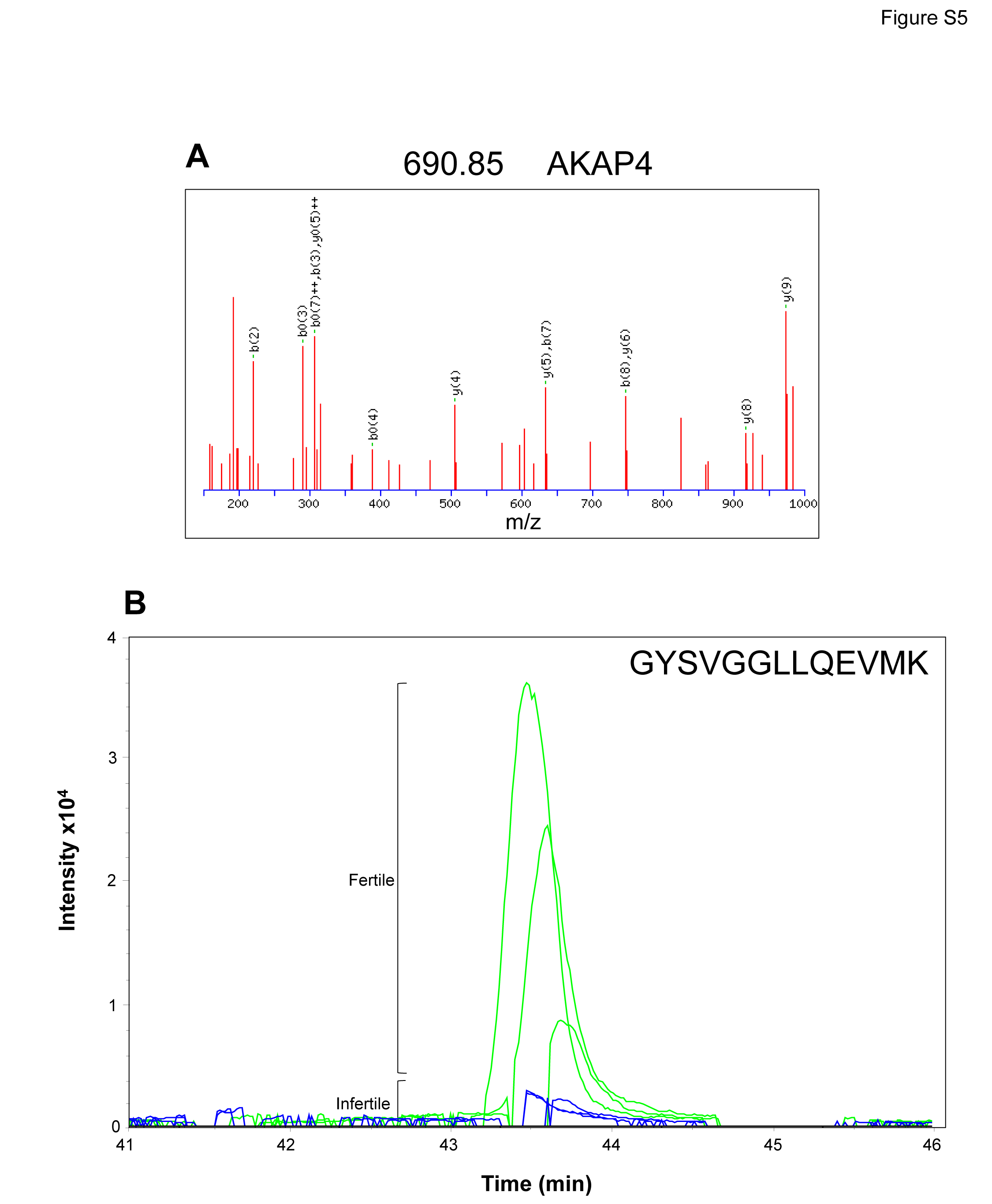

Supplement: Figure S5 — A-kinase anchoring protein AKAP4. (A) Tandem mass spectra profile of the monoisotopic ion precursor m/z 690.85 (2+) together with the annotated y and b ion series. The tryptic peptide sequence obtained, GYSVGGLLQEVMK, matched to the protein AKAP4. (B) Extracted ion chromatograms (EIC) from samples collected using MS-only precursor scans (1 Hz, 50–2000 Da). A significant difference (p = 0.029) is demonstrated when the EIC from 3 independent semen samples from a fertile donor (green traces) are overlayed with 3 independent samples from an infertile patient (blue traces) whose spermatozoa could not bind to the ZP. (TIF) [file pone.0050851.s005.tif]

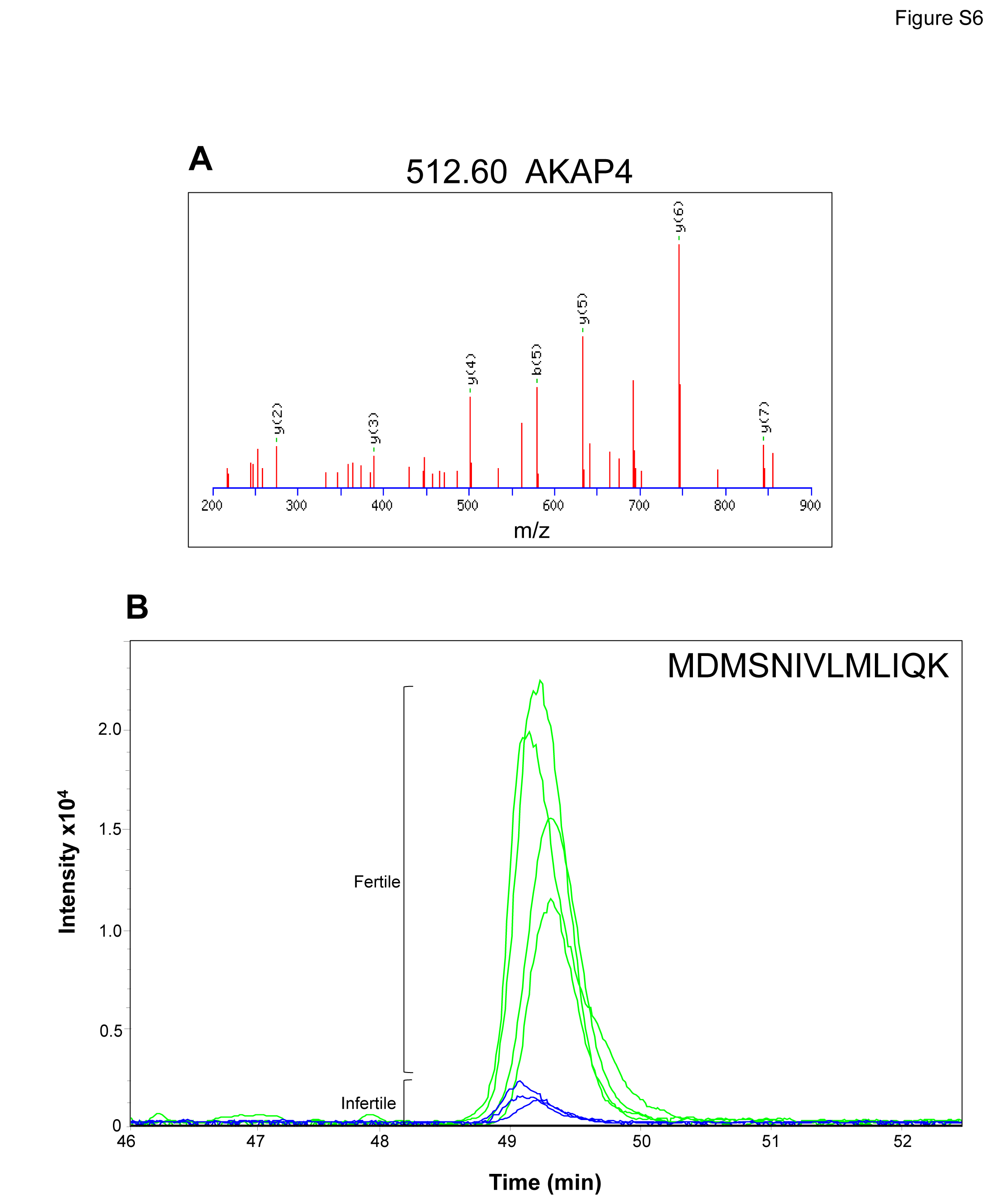

Supplement: Figure S6 — A-kinase anchoring protein AKAP4. (A) Tandem mass spectra profile of the monoisotopic ion precursor m/z 512.60 (3+) together with the annotated y and b ion series. The tryptic peptide sequence obtained, MDMSNIVLMLIQK, matched to the protein AKAP4. (B) Extracted ion chromatograms (EIC) from samples collected using MS-only precursor scans (1 Hz, 50–2000 Da). A significant difference (p<0.001) is demonstrated when the EIC from 3 independent semen samples from a fertile donor (green traces) are overlayed with 3 independent samples from an infertile patient (blue traces) whose spermatozoa could not bind to the ZP. (TIF) [file pone.0050851.s006.tif]

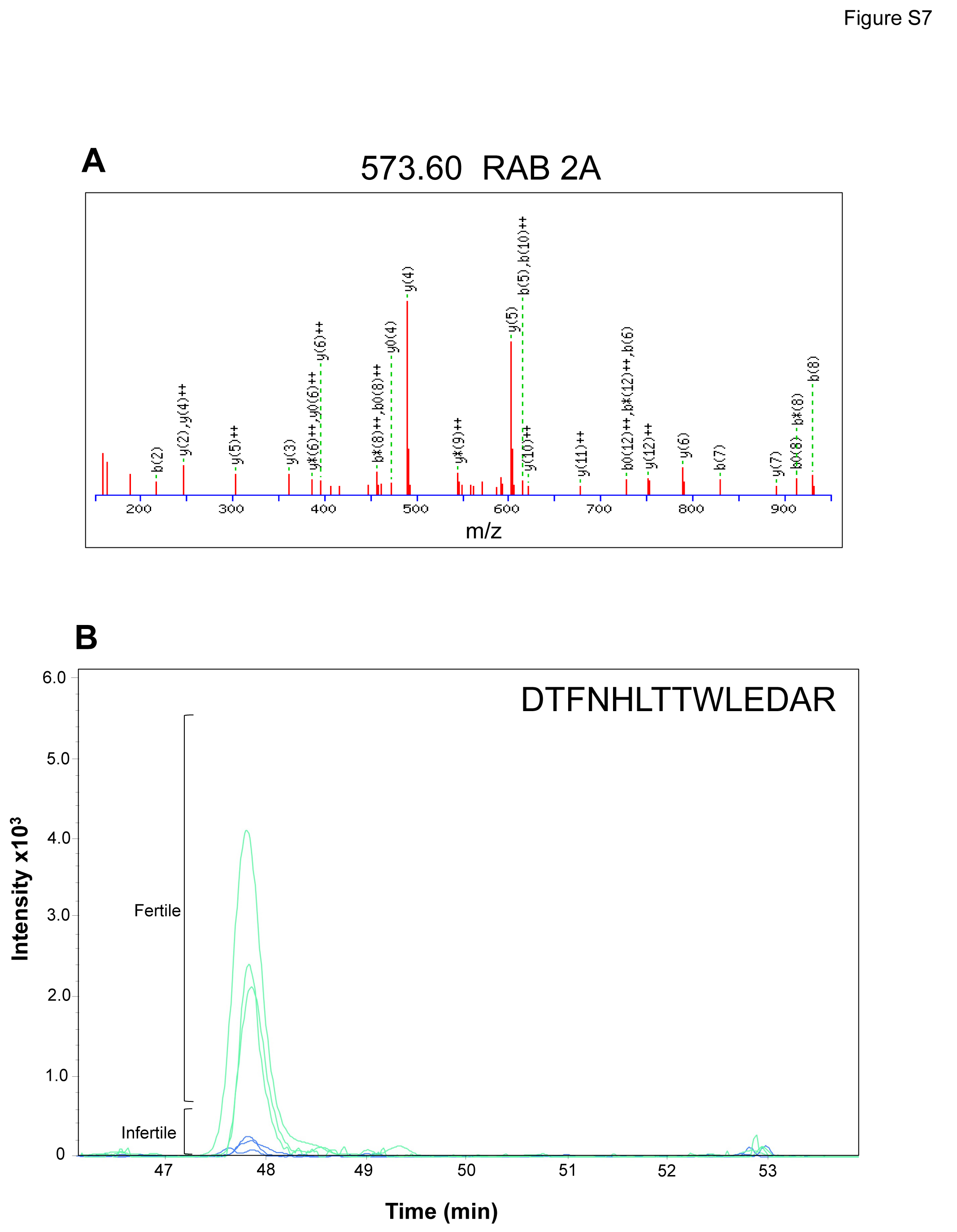

Supplement: Figure S7 — Ras related protein, Rab2A. (A) Tandem mass spectra profile of the monoisotopic ion precursor m/z 573.60 (3+) together with the annotated y and b ion series. The tryptic peptide sequence obtained, DTFNHLTTWLEDAR, matched to the protein, Ras related protein, Rab2A. (B) Extracted ion chromatograms (EIC) from samples collected using MS-only precursor scans (1 Hz, 50–2000 Da). A significant difference (p<0.001) is demonstrated when the EIC from 3 independent semen samples from a fertile donor (green traces) are overlayed with 3 independent samples from an infertile patient (blue traces) whose spermatozoa could not bind to the ZP. (TIF) [file pone.0050851.s007.tif]

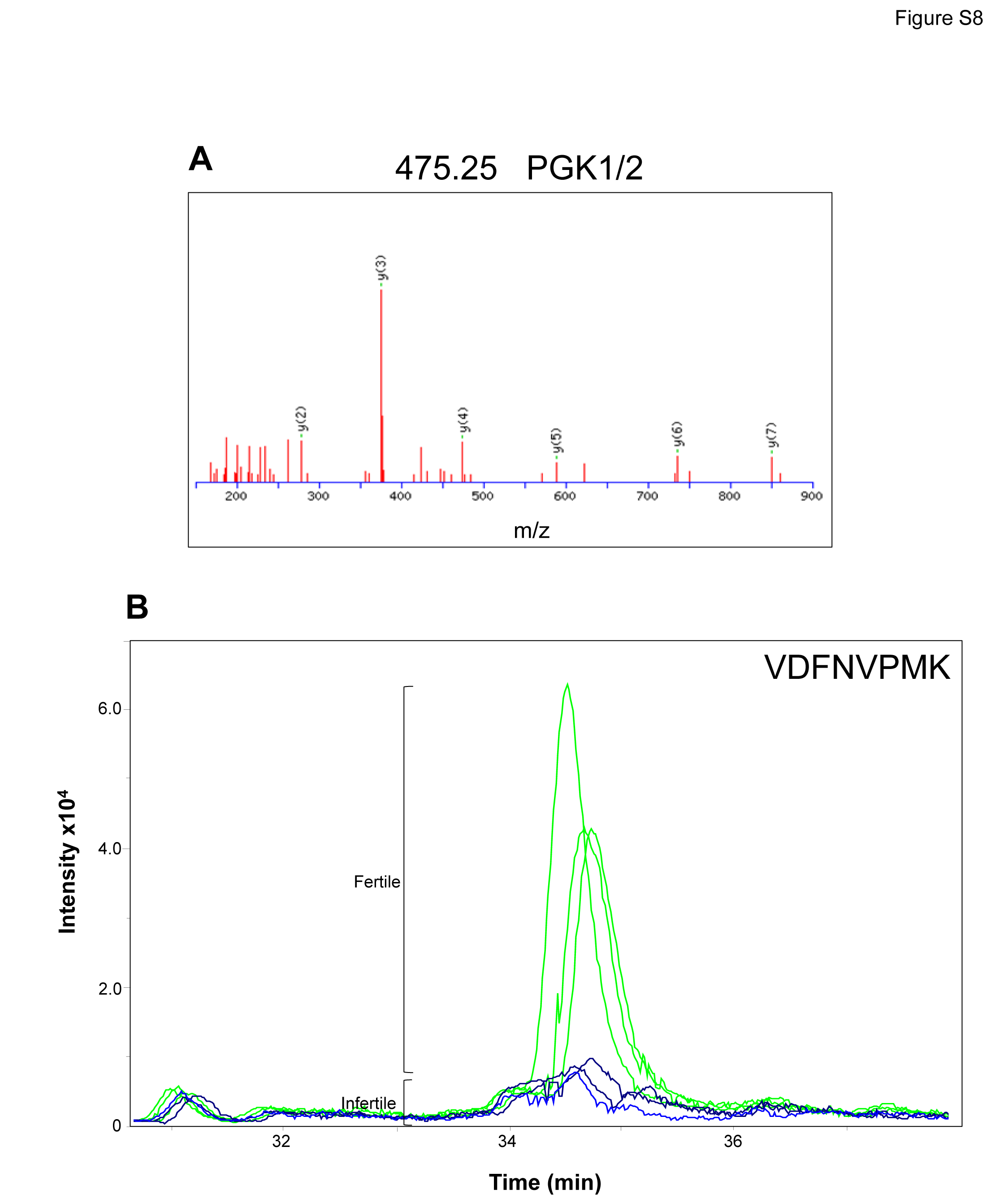

Supplement: Figure S8 — Phosphoglycerate kinase, PGK-1/2. (A) Tandem mass spectra profile of the monoisotopic ion precursor m/z 475.25 (3+) together with the annotated y and b ion series. The tryptic peptide sequence obtained, VDFNVPMK, matched to the protein PGK-1/2. (B) Extracted ion chromatograms (EIC) from samples collected using MS-only precursor scans (1 Hz, 50–2000 Da). A significant difference (p<0.001) is demonstrated when the EIC from 3 independent semen samples from a fertile donor (green traces) are overlayed with 3 independent samples from an infertile patient (blue traces) whose spermatozoa could not bind to the ZP. (TIF) [file pone.0050851.s008.tif]

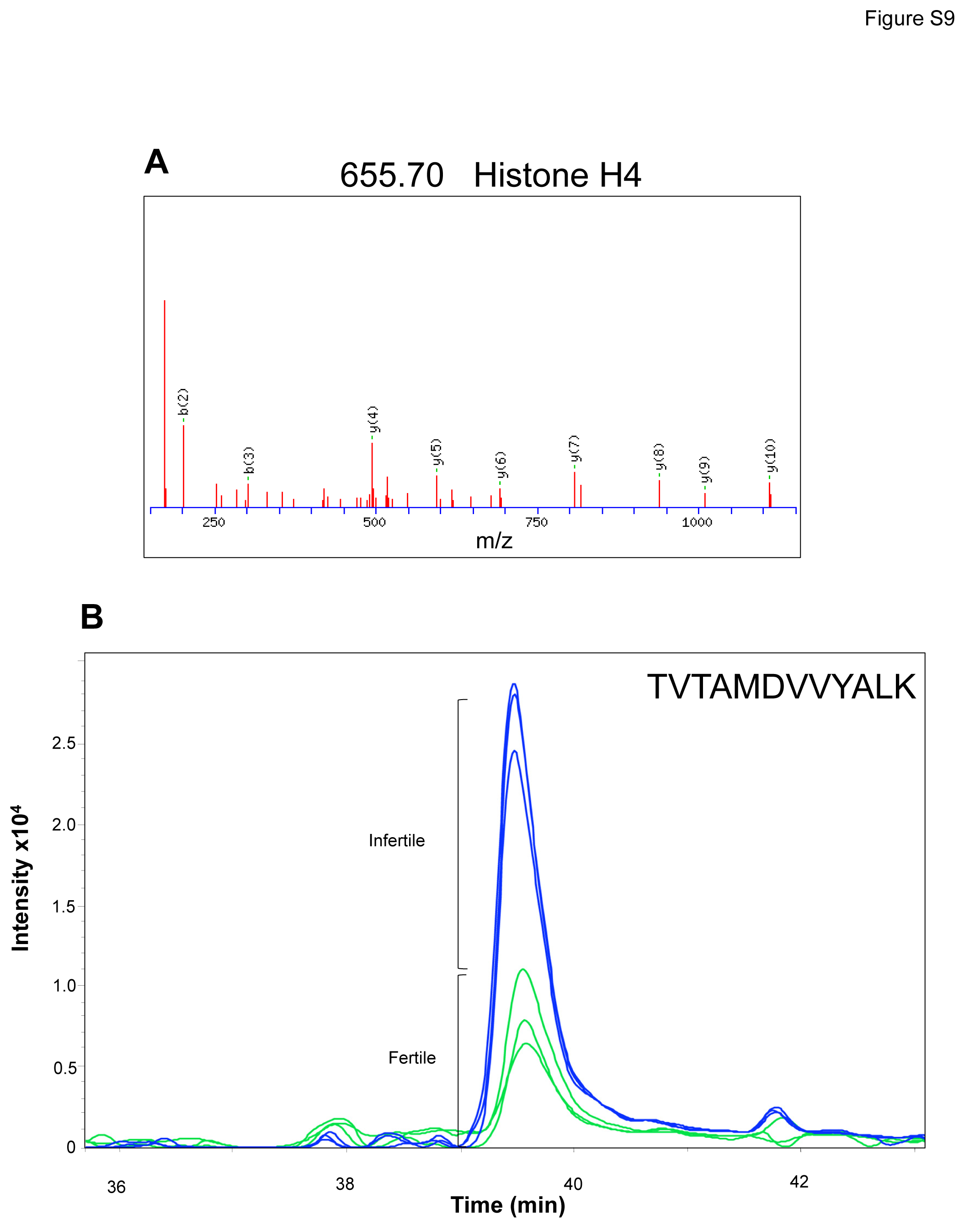

Supplement: Figure S9 — Histone H4. (A) Tandem mass spectra profile of the monoisotopic ion precursor m/z 655.70 (2+) together with the annotated y and b ion series. The tryptic peptide sequence obtained, TVTAMDVVYALK, matched to the protein, Histone H4. (B) Extracted ion chromatograms (EIC) from samples collected using MS-only precursor scans (1 Hz, 50–2000 Da). A significant difference (p<0.001) is demonstrated when the EIC from 3 independent semen samples from a fertile donor (green traces) are overlayed with 3 independent samples from an infertile patient (blue traces) whose spermatozoa could not bind to the ZP. (TIF) [file pone.0050851.s009.tif]
