# Supplementary material for: The Molecular Chaperone HSPA2 Plays a Key Role in Regulating the Expression of Sperm Surface Receptors That Mediate Sperm-Egg Recognition
Source: PLoS One. 2012 Nov 29;7(11):e50851. doi: 10.1371/journal.pone.0050851 (PMC3510172; doi:10.1371/journal.pone.0050851)
Supplement: Table S1 — Protein identities of 200 kDa complex obtained via MS/MS analysis. (DOC) [file pone.0050851.s010.doc]

Table S1: Protein identities of 200kDa complex obtained via MS/MS analysis

| **Protein (symbol)** | **UniProt accession number** | **Molecular weight (kDa)** | **Number matched peptides** | **Peptide sequence** | **Mascot score for individual peptides** | **Overall Mascot score** |
| --- | --- | --- | --- | --- | --- | --- |
| Arylsulfatase (ARSA) | P15289 | 53,772 | 6 | MGMYPGVLVPSSR AQLDAAVTFGPSQVAR YMAFAHDLMADAQR GGLPLEEVTVAEVLAAR QSLFFYPSYPDEVR DPGENYNLLGGVAGATPEVLQALK | 74 148 74 122 63 101 | 425 |
| Sperm Adhesion Molecule 1 isoform 1 (SPAM1) | Q5D1J4 | 58,358 | 3 | IVFTDQVLK SPLPVFAYTR AGKDFLVETIK | 40 47 39 | 63 |
| Heat Shock-Related 70kDa Protein 2 (HSPA2) | P54652 | 69,978 | 19 | GTLEPVEK LLQDFFNGK FDLTGIPPAPR VEIIANDQGNR DAGTITGLNVLR FEELNADLFR MKEIAEAYLGGK NALESYTYNIK GQIQEIVLVGGSTR ARFEELNADLFR TTPSYVAFTDTER TFFPEEISSMVLTK IINEPTAAAIAYGLDK NQVAMNPTNTIFDAK STAGDTHLGGEDFDNR IINEPTAAAIAYGLDKK LDKGQIQEIVLVGGSTR VHSAVITVPAYFNDSQR NVLIFDLGGGTFDVSILTIEDGIFEVK | 40 41 58 76 93 87 67 77 105 73 95 76 118 104 68 87 71 101 118 | 940 |
